# Near-final adult height after GnRHa plus growth hormone in central precocious puberty: a single-center cohort study and predicted adult height validation audit

**DOI:** 10.3389/fendo.2026.1885575

**Published:** 2026-09-18

**Authors:** Peili Jin, Duomei Wang, Minyan Qian, Huimin Zhang, Chang Liu, Rongwei Yang

**Affiliations:** 1 Jiaxing Maternity and Child Health Care Hospital, Jiaxing, Zhejiang, China; 2 Medical Affairs Department, GeneScience Pharmaceuticals, Changchun, Jilin, China

**Keywords:** central precocious puberty, confounding by indication, gonadotropin-releasing hormone analog, growth hormone, near-final adult height, observational methods, predicted adult height, validation audit

## Abstract

**Background:**

Whether adjunctive recombinant human growth hormone (rhGH) improves near-final adult height (NFAH) during gonadotropin-releasing hormone analog (GnRHa) therapy for central precocious puberty (CPP) remains uncertain, and predicted adult height (PAH) may reflect treatment selection and prognosis.

**Methods:**

We analyzed a single-center retrospective cohort of 235 children with CPP who initiated GnRHa between January 2018 and December 2020. The primary endpoint was chart-recorded NFAH in the female-only maturity-ascertained cohort (n = 91); the criterion-confirmed all-sex set included 94 patients. The primary adjusted model (M2) included treatment, baseline age, body mass index (BMI), IGF-1, BA–CA, and baseline height; baseline bone age was not entered separately because bone age = chronological age + BA–CA. Missing baseline covariates were handled using 20 imputations by chained equations. Height gain and change in height standard deviation score (SDS) from baseline to 24 months were secondary outcomes. Results: Mean NFAH was 160.17 cm with GnRHa alone and 161.87 cm with GnRHa + rhGH. The adjusted M2 association was +2.26 cm (95% CI 0.12 to 4.40); the chart-PAH-conditioned M3 estimate was +0.58 cm (−1.60 to 2.75) and was treated as diagnostic. At 24 months, M2 estimates were +3.23 cm (2.00 to 4.46) for height gain, +3.24 cm (2.03 to 4.46) in analysis of covariance (ANCOVA) of attained height, and +0.46 SDS (0.27 to 0.66). In the criterion-confirmed female bone-age cohort, M2 estimates were +0.00 years (−0.27 to 0.27) for bone age at 24 months and −0.14 years (−0.30 to 0.03) for bone-age progression.

**Conclusions:**

Adjunctive rhGH was associated with greater growth over 24 months and a positive, imprecisely estimated NFAH difference. Because this was an observational study, ascertainment criteria were heterogeneous, and the NFAH estimate was sensitive to PAH adjustment and criterion restriction, the adult-height finding should be interpreted cautiously.

## 1 Introduction

Central precocious puberty (CPP) accelerates pubertal progression and skeletal maturation, narrowing the window for linear growth and reducing the projected adult height of affected children, particularly girls (1–4). Gonadotropin-releasing hormone analogs (GnRHa) are the standard of care: they suppress the hypothalamic-pituitary-gonadal axis and help restore alignment between chronological and biological age, thereby delaying premature epiphyseal fusion (5–7). Secular changes in pubertal timing and increasing CPP diagnoses have further sharpened clinical interest in long-term outcomes (8–11). However, meta-analyses of GnRHa monotherapy have repeatedly documented residual height deficits relative to genetic potential, especially among children with low baseline predicted adult height (PAH) (12–14). To address this shortfall, several centers have added recombinant human growth hormone (rhGH) to GnRHa therapy, motivated by the rationale that rhGH may increase residual linear growth potential while skeletal maturation is suppressed by ongoing GnRHa exposure (15–21).

A Single-arm follow-up study has also reported improvement in final height after GnRHa monotherapy (22). Observational studies of adjunctive rhGH and meta-analyses published from 2016 to 2025 have reported short-term height gains of approximately 1.5 to 3 cm at 12–24 months in GnRHa + rhGH versus GnRHa-only cohorts (15, 17, 18, 23, 24). These benefits have varied in magnitude, have been sensitive to follow-up duration, and have not consistently translated into improvements in near-final adult height (NFAH), the endpoint most relevant to decisions about adding rhGH. Where NFAH has been reported, sample sizes were small and confounding control was typically restricted to age, sex, and baseline height (16, 19–21). Final-height evidence in CPP therefore remains limited in both extent and statistical depth.

A specific form of confounding has been underemphasized in this literature. Baseline PAH, usually derived from bone age via the Bayley–Pinneau method (25, 26), is attractive because it summarizes baseline height prognosis and may inform escalation from GnRHa monotherapy to add-on rhGH. However, PAH is not automatically a valid deconfounder. If PAH is poorly calculated, recorded after clinical decision-making, weakly prognostic, or more strongly associated with treatment than with outcome, conditioning on it can amplify bias or condition on treatment intent (27). We therefore treat PAH as a variable requiring validation rather than as a prespecified causal solution.

If baseline PAH captures a substantial part of indication-driven selection, sequential adjustment for standard covariates, then baseline height, then baseline PAH would be expected to attenuate the apparent rhGH association with NFAH. A bone-age-based sensitivity endpoint can also help assess whether a similar attenuation pattern is observed for skeletal maturation, an outcome not expected to be the primary target of adjunctive rhGH in children already receiving GnRHa. If the NFAH association attenuates while the 24-month association persists under the same adjustment cascade, the pattern would support the interpretation that rhGH is associated with higher on-treatment height but not with a durable adult-height advantage after baseline prognosis is accounted for.

We evaluated this question in a single-center retrospective cohort of 235 children with CPP. Ninety-four patients met at least one prespecified, sex-appropriate NFAH ascertainment criterion; the prespecified primary analysis comprised 91 girls. The objective was to estimate the 24-month and NFAH associations, use the PAH-free M2 model as the primary adjusted NFAH analysis, and retain chart-recorded PAH only as a diagnostic covariate because its formula and timing were not uniformly documented.

## 2 Methods

### 2.1 Study design, setting, and participants

This study was designed as a single-center retrospective comparative cohort study using de-identified clinical records of children with central precocious puberty (CPP) consecutively initiated on GnRHa therapy at the participating tertiary pediatric endocrinology center between January 2018 and December 2020. CPP diagnosis required pubertal onset before 8 years in girls or 9 years in boys, progressive clinical pubertal development, accelerated skeletal maturation, and biochemical activation of the hypothalamic-pituitary-gonadal axis on a GnRH stimulation test (peak luteinizing hormone ≥5 IU/L). Cases with documented peripheral precocious puberty, congenital adrenal hyperplasia, exogenous sex-steroid exposure, a known central nervous system tumor, or another organic cause requiring disease-specific management were excluded when identified in the clinical workup. The locked analytic cohort comprised 235 patients (110 GnRHa + rhGH, 125 GnRHa only); the cohort was locked on 16 April 2026, and a separate ethics amendment approved on the same date authorized the subsequent NFAH chart abstraction conducted under a prespecified protocol. Availability of either a 24-month height measurement or an NFAH record was treated as an ascertainment status rather than an entry criterion, so that missing-data sensitivity analyses could be conducted on the full locked cohort. The study was approved by the participating hospital’s Ethics Committee; the requirement for written informed consent was waived for the retrospective use of de-identified clinical records. All procedures were performed in accordance with the Declaration of Helsinki.

Two data sources were used. The locked longitudinal dataset provided baseline characteristics and the 0–24-month height, weight, and insulin-like growth factor 1 (IGF-1) series. A subsequent supplementary chart abstraction provided bone age at 12 and 24 months, the last recorded bone age, age and date at NFAH ascertainment, menarcheal date, adverse-event fields, and adult-height and predicted-adult-height entries. The NFAH height, ascertainment criterion, age/date, bone-age, and PAH fields were verified record by record against the original clinical records. Cross-source quality-control fields classified baseline height as concordant in 235/235 record pairs and baseline bone age in 211/235 record pairs (concordance was assessed as agreement between sources, including agreement that a value was absent); these are reconciliation counts, not non-missing-data denominators. In the analysis-ready extract, baseline height was available for 233/235 patients and baseline bone age for 183/235 (100/125 GnRHa only and 83/110 GnRHa + rhGH); baseline bone age was available for 70/94 patients in the criterion-confirmed NFAH set. **Table 1** reports the cell-specific denominator for every incompletely observed baseline variable.

**Table 1:** Baseline characteristics of the locked analytic cohort and the criterion-confirmed NFAH set.

Variable	Whole cohort (N = 235)	GnRHa only	GnRHa + rhGH	Criterion-confirmed NFAH (n = 94), all sexes	NFAH – GnRHa only	NFAH – GnRHa + rhGH
N	235	125	110	94	53	41
Female, n (%)	209 (88.9%)	122 (97.6%)	87 (79.1%)	91 (96.8%)	51 (96.2%)	40 (97.6%)
Age (years)	9.2 (1.1) [n = 233]	8.7 (0.9) [n = 125]	9.9 (1.0) [n = 108]	9.1 (1.0) [n = 93]	8.8 (0.9) [n = 53]	9.6 (0.8) [n = 40]
Height (cm)	138.1 (6.9) [n = 233]	137.0 (6.6) [n = 124]	139.3 (7.0) [n = 109]	137.8 (6.3) [n = 94]	137.7 (6.3) [n = 53]	137.8 (6.4) [n = 41]
BMI (kg/m²)	17.6 (2.4) [n = 174]	17.8 (2.3) [n = 93]	17.4 (2.6) [n = 81]	17.1 (2.1) [n = 68]	17.6 (2.0) [n = 39]	16.6 (2.1) [n = 29]
IGF-1 (ng/mL)	299 [232, 381] [n = 137]	270 [219, 352] [n = 50]	328 [241, 399] [n = 87]	287 [230, 360] [n = 59]	299 [231, 353] [n = 25]	284 [230, 361] [n = 34]
Bone age (years)	11.0 (1.2) [n = 183]	10.5 (1.0) [n = 100]	11.7 (1.0) [n = 83]	10.8 (1.0) [n = 70]	10.4 (1.0) [n = 42]	11.3 (0.9) [n = 28]
BA − CA (years)	1.81 (0.76) [n = 181]	1.89 (0.68) [n = 100]	1.72 (0.85) [n = 81]	1.72 (0.64) [n = 69]	1.78 (0.61) [n = 42]	1.64 (0.69) [n = 27]
Chart-recorded baseline PAH (cm)	158.7 (1.7) [n = 95]	158.0 (1.5) [n = 53]	159.6 (1.5) [n = 42]	158.7 (1.7) [n = 94]	158.0 (1.5) [n = 53]	159.7 (1.6) [n = 41]
Recalculated BP-PAH (calculable subset, cm)	145.2 (5.9) [n = 138]	146.1 (6.4) [n = 89]	143.5 (4.4) [n = 49]	146.3 (6.1) [n = 59]	148.1 (6.7) [n = 36]	143.5 (3.8) [n = 23]

Values are mean (standard deviation), n (%), or median [interquartile range] as appropriate. Every incompletely observed continuous-variable cell includes its non-missing denominator. BMI, body mass index; BA, bone age; CA, chronological age; IGF-1, insulin-like growth factor 1; PAH, predicted adult height; BP, Bayley–Pinneau. The criterion-confirmed NFAH set includes 94 patients; the primary NFAH analysis is restricted to 91 girls. Chart-recorded PAH was available in 95 case records; the NFAH columns summarize the 94 patients who also met a prespecified, sex-appropriate maturity criterion. Recalculated BP-PAH is shown only for records with the inputs needed for that calculation and is descriptive.

The electronic abstraction dataset retained standardized auxologic and laboratory variables needed for the prespecified causal analysis. Tanner stage, peak follicle-stimulating hormone (FSH), luteinizing hormone (LH)/FSH ratio, estradiol or testosterone, pelvic ultrasonography, ovarian or uterine dimensions, and testicular volume were reviewed clinically during routine diagnosis when available; however, these fields were not uniformly encoded in the locked de-identified extract and therefore were not used as covariates, limiting detailed phenotypic comparison between treatment arms.

### 2.2 Treatment groups and follow-up

Treatment group was defined from clinical treatment records as the regimen actually administered at the index visit. Children in the GnRHa-only arm received depot triptorelin 80–100 µg/kg every 28 days by subcutaneous injection; dose adjustment during follow-up was made by the treating pediatric endocrinologist according to pubertal suppression, growth tempo, bone-age progression, and stimulated or basal gonadotropin results from clinically repeated testing. Children in the GnRHa + rhGH arm received the same GnRHa schedule plus daily recombinant human growth hormone subcutaneously at 0.10–0.15 IU/kg/day, corresponding approximately to 0.033–0.050 mg/kg/day or 0.23–0.35 mg/kg/week using the conventional 3 IU = 1 mg conversion. The decision to escalate from GnRHa monotherapy to combined therapy was made by the treating endocrinologist on clinical grounds, most commonly a PAH judged insufficient relative to mid-parental target height, slow growth under suppression, or concern that residual growth potential would not achieve the family-specific target. Treatment discontinuation was individualized according to chronological age, bone age, pubertal suppression, growth velocity, and family preference; data on structured adherence, injection interruptions, and exact discontinuation dates were not consistently available in the retrospective extract.

### 2.3 Endpoints

The primary endpoint was near-final adult height (NFAH), measured in centimeters and ascertained by any one of five prespecified criteria applied in priority order: (D) a record explicitly labeled as adult or final height; (E) manual confirmation from the clinical record; (B) at least 2 years after menarche in girls; (C) two consecutive height measurements differing by < 1 cm over 6 months; and (A) either chronological age ≥14 years in girls or ≥16 years in boys, or a last recorded bone age ≥14 years in girls or ≥16 years in boys. Bone ages shown in **Table 2** were recorded at baseline, at 24 months, and at the last available bone-age assessment; they were not assumed to have been obtained on the date of NFAH ascertainment. We used NFAH because a small amount of residual growth can remain in some adolescents meeting criteria A or B. The criterion-confirmed set comprised 94 patients (91 girls, 3 boys), and the prespecified primary analysis was female-only (n = 91). The secondary height endpoint was analyzed as both gain from baseline to 24 months and attained height, with baseline height included as a covariate. Bone-age sensitivity analyses used the criterion-confirmed female NFAH cohort and the supplementary chart abstraction: 83 girls had bone age at 24 months, and 63 had both baseline and 24-month bone age. These bone-age denominators are separate from the 78/77-girl 24-month height cohorts.

**Table 2:** Criterion-specific maturity profile for NFAH ascertainment.

NFAH ascertainment criterion	n	Age at baseline, y	Age at NFAH, y	Interval baseline to NFAH, y	Last on-protocol height visit, mo (a)	Bone age at baseline, y	Bone age at 24 mo, y	Last recorded bone age, y (b)
A. Chronological-age or last-recorded-bone-age threshold	22	9.6 (0.8) [n = 21]	15.0 (0.8) [n = 22]	5.43 (0.64) [n = 21]	17.7 (5.3) [n = 22]	11.2 (1.0) [n = 12]	12.4 (1.1) [n = 20]	13.9 (1.1) [n = 20]
B. Menarche ≥2 years (girls)	35	8.8 (0.9) [n = 35]	13.9 (1.0) [n = 35]	5.15 (0.47) [n = 35]	18.6 (6.1) [n = 35]	10.4 (1.1) [n = 29]	11.6 (1.1) [n = 32]	13.0 (1.2) [n = 32]
C. Height change < 1 cm over 6 months	29	9.1 (1.1) [n = 29]	14.1 (1.2) [n = 29]	5.04 (0.50) [n = 29]	18.5 (6.6) [n = 29]	10.9 (1.0) [n = 23]	12.0 (1.1) [n = 27]	13.3 (1.1) [n = 27]
D. Recent adult/final-height record	6	9.5 (0.6) [n = 6]	14.9 (0.6) [n = 6]	5.39 (0.42) [n = 6]	20.5 (4.8) [n = 6]	11.1 (0.4) [n = 4]	11.7 (0.8) [n = 6]	13.1 (0.9) [n = 6]
E. Manual chart confirmation	2	10.5 (1.0) [n = 2]	15.2 (1.2) [n = 2]	4.71 (0.20) [n = 2]	24.0 (0.0) [n = 2]	11.2 (0.6) [n = 2]	12.6 [n = 1]	14.5 [n = 1]
All criterion-confirmed NFAH	94	9.1 (1.0) [n = 93]	14.3 (1.1) [n = 94]	5.19 (0.53) [n = 93]	18.6 (5.9) [n = 94]	10.8 (1.0) [n = 70]	11.9 (1.1) [n = 86]	13.4 (1.1) [n = 86]

Values are mean (standard deviation) [available n] unless otherwise indicated. NFAH, near-final adult height. The interval from baseline to NFAH is age at NFAH ascertainment minus baseline chronological age. (a) Last on-protocol height visit is the last recorded visit within the 0–24-month treatment-observation series; it is censored at 24 months and is not the interval to NFAH. (b) Bone ages are presented at baseline, 24 months, and the last available assessment; none is assumed to have been measured on the NFAH ascertainment date. Criterion A could be met through either the sex-specific chronological-age threshold or the sex-specific last-recorded-bone-age threshold, so its mean bone age is not constrained to exceed the cutoff. Treatment-discontinuation dates were not uniformly available.

The four male records with an NFAH-height entry were reviewed against the sex-specific criteria. One met criterion C; two met criterion A, one through the chronological-age threshold and one through the last-recorded-bone-age threshold; and one did not have documentation of any prespecified maturity criterion and was excluded from the criterion-confirmed all-sex set. This review changed the all-sex sensitivity cohort from 95 to 94 but did not alter the female-only primary cohort (n = 91).

### 2.4 Baseline covariates and predicted adult height

Baseline covariates were recorded from the electronic medical record by trained clinical investigators using a structured chart-abstraction form. The standard M1 covariate set comprised age (decimal years), body mass index (BMI, kg/m²), serum insulin-like growth factor 1 (IGF-1, ng/mL), and the bone-age-minus-chronological-age gap (BA–CA); sex was additionally included only in all-sex sensitivity analyses and was omitted from female-only models because it was constant. Baseline height was deliberately excluded from M1 and introduced once, at M2. Baseline bone age was not entered as a separate term because BA–CA = bone age − chronological age; simultaneous entry of all three variables creates exact linear dependence. Pediatric IGF-1 varies with age and pubertal stage, and continuous reference intervals have been reported for Chinese children (28). The locked extract did not consistently retain assay-specific reference ranges or puberty-adjusted IGF-1 SDS, so models used the chart-recorded concentrations and interpreted them cautiously. Parental heights were available in 81 NFAH records but were not part of the prespecified adjustment set. Standardized height and BMI values were based on the 2009 Chinese growth charts (29). Height standard deviation scores (SDS) were computed for girls with the lambda-mu-sigma (LMS) method, z = ((X/M)^L − 1)/(L × S), using linearly interpolated girls’ height-for-age parameters at exact decimal age. Change in height SDS from baseline to 24 months (Δ height SDS) was reported as a scale-free companion to absolute height gain.

Two PAH variables were distinguished. Chart-recorded pretreatment PAH was taken directly from the clinical record. The chart field was non-missing in exactly the same 95 records that contained an NFAH-height entry—94 criterion-confirmed records plus the one excluded male record—and was absent from the other 140 locked-cohort records. This one-to-one pattern suggests that PAH was populated during the same NFAH-focused retrospective abstraction rather than being a uniformly available baseline field. In addition, the field did not uniformly retain its calculation formula, source table, entry timestamp, or maturity-category definition. It was therefore not selected for primary causal adjustment. In the female NFAH cohort, it was weakly correlated with baseline bone age (r = 0.07, n = 68) and more strongly associated with treatment assignment (r = 0.48). Recalculated Bayley–Pinneau PAH (BP-PAH) was available for a subset of patients for whom it could be calculated and was used only for a method-comparison audit. The variance inflation factor (VIF) reported for chart-recorded PAH describes its relationship with the other M3 covariates; it does not address the exact identity among chronological age, bone age, and BA–CA, which was handled by excluding bone age as a separate model term.

PAH assessment included per-arm distributions, correlations with baseline height, bone age, BA–CA, treatment, and NFAH, and calibration against observed NFAH. Because chart-recorded PAH may encode both prognosis and the clinical decision to add rhGH, all PAH-conditioned analyses are described as diagnostic rather than primary.

### 2.5 Statistical analysis

All primary regressions were ordinary least squares linear models fitted in the female-only analytic cohorts. With Y denoting NFAH (n = 91), height gain from baseline to 24 months (n = 77), change in height SDS (n = 77), or attained height at 24 months (n = 78), the complete formulas were: M0, Y ~ treatment; M1, Y ~ treatment + baseline age + BMI + IGF-1 + BA–CA; M2, M1 + baseline height; and M3, M2 + chart-recorded baseline PAH. Thus, baseline height is not included in M1 and is added once in M2. Baseline bone age is not entered separately. A design-matrix audit of simultaneous age, bone age, and BA–CA entry confirmed rank 6 for 7 columns and identified bone age as the aliased column under the recorded term order. The reported M0, M1, M2, and M3 matrices were full rank, with ranks 2/2, 6/6, 7/7, and 8/8, respectively, and no variable was automatically omitted in any imputed dataset. Missing baseline covariates were handled using 20 multivariate imputations by chained equations (30, 31). The imputation model included treatment, the observed outcome, baseline age, height, BMI, IGF-1, bone age, and chart PAH; outcome values were never imputed, and BA–CA was recalculated after imputation. Estimates were pooled with Rubin’s rules and Barnard–Rubin small-sample degrees of freedom. M2 was the prespecified primary PAH-free adjusted analysis; M3 was diagnostic. Complete coefficients, standard errors, matrix rank, VIFs, condition indices, model-fit statistics, and residual diagnostics are reported in **Supplementary Table S1–S2**.

Sensitivity analyses comprised complete-case refits, criterion-restricted NFAH analyses excluding criterion E and then retaining only criteria A–C, the all-sex criterion-confirmed cohort, the height-gain and height-SDS outcomes, and analysis of covariance (ANCOVA) of attained 24-month height with baseline height as a covariate. Treatment-coefficient changes were also reported after adding each covariate sequentially within the same analytic sample. Exploratory PAH-stratified and causal-forest displays, following the heterogeneous-treatment-effect framework of Wager and Athey (32), were retained only as descriptive analyses and were not used to support the primary conclusion.

Three cohort definitions supported the analysis. The primary NFAH cohort comprised 91 girls who met a prespecified maturity criterion. The criterion-confirmed all-sex sensitivity cohort comprised 94 patients (91 girls and 3 boys). The 24-month cohort included 91 patients with an observed 24-month height; parallel female-only analyses used 78 girls for attained height and 77 for height gain because one lacked baseline height. Cohort flow and ascertainment counts are summarized in **Figure 1** and **Table 3**. All tests were two-sided, and 95% confidence intervals are reported.

**Figure 1:**
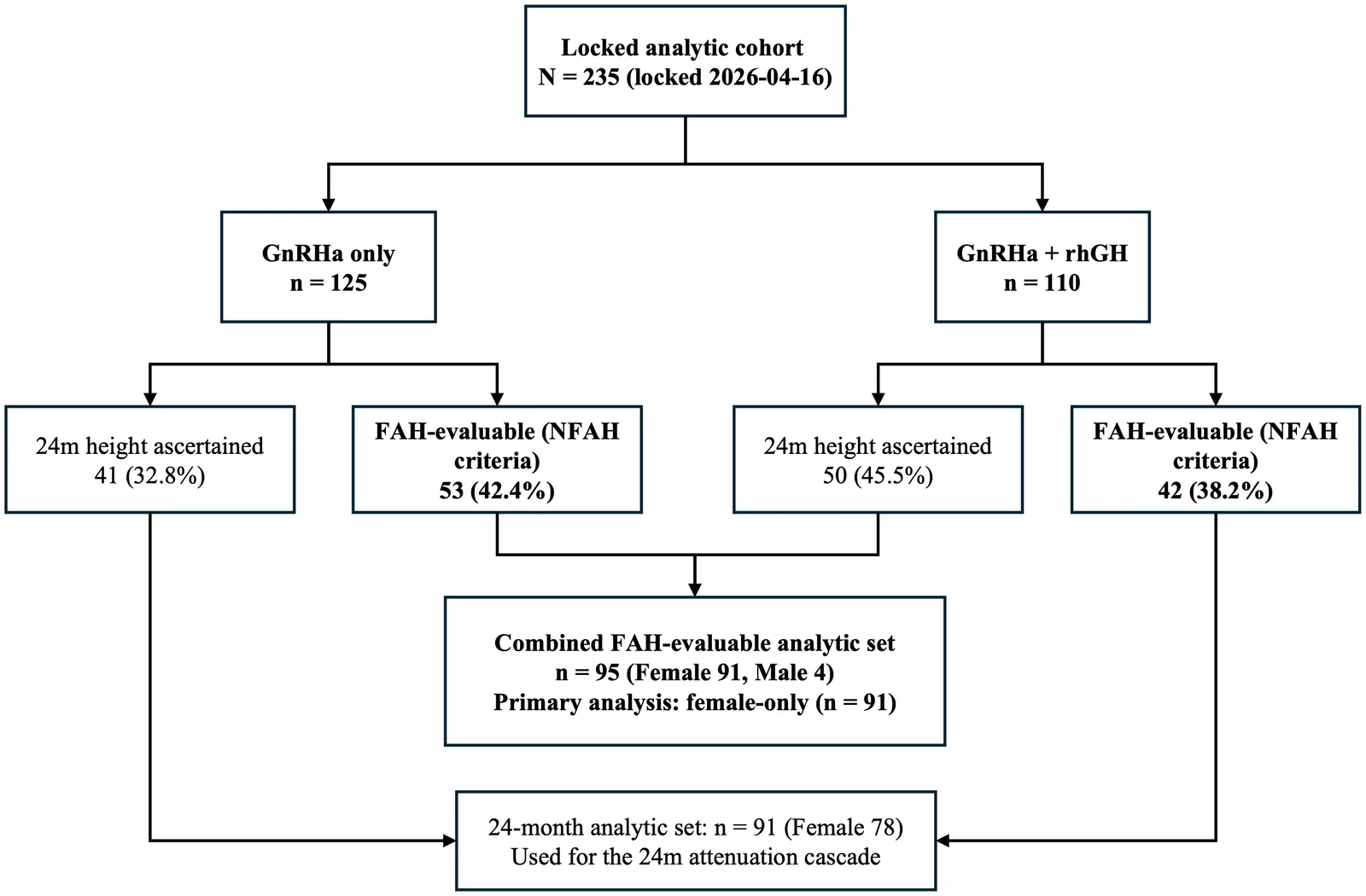
Cohort flow and NFAH ascertainment. The locked cohort (N = 235) comprised 125 GnRHa-only and 110 GnRHa + rhGH patients. Twenty-four-month height was available for 41 and 50 patients, respectively. The criterion-confirmed NFAH set comprised 53 and 41 patients (n = 94; 91 girls, 3 boys); the primary NFAH analysis was female-only (n = 91). The female-only 24-month analyses included 78 girls for attained height and 77 for height gain. The footer shows the NFAH criterion distribution.

**Table 3:** Cohort flow and NFAH-ascertainment-criterion distribution.

Stage	All (%)	GnRHa only (%)	GnRHa + rhGH (%)
Locked analytic cohort	235 (100.0%)	125 (100.0%)	110 (100.0%)
24-month height ascertained	91 (38.7%)	41 (32.8%)	50 (45.5%)
Criterion-confirmed NFAH	94 (40.0%)	53 (42.4%)	41 (37.3%)
NFAH ascertainment criteria	Not applicable	Not applicable	Not applicable
A. Chronological-age or last-recorded-bone-age threshold	22 (23.4%)	Not applicable	Not applicable
B. Menarche ≥2 years (girls)	35 (37.2%)	Not applicable	Not applicable
C. Height change < 1 cm over 6 months	29 (30.9%)	Not applicable	Not applicable
D. Recent adult/final-height record	6 (6.4%)	Not applicable	Not applicable
E. Manual chart confirmation	2 (2.1%)	Not applicable	Not applicable

Percentages in the first three rows are relative to each treatment arm’s locked-cohort denominator. Criterion percentages are relative to the criterion-confirmed NFAH set (n = 94). Criterion A is satisfied by either the sex-specific chronological-age threshold or the sex-specific last-recorded-bone-age threshold. One boy with an NFAH-height entry but no documented prespecified maturity criterion was excluded from this set; the female-only primary cohort was unchanged. NFAH, near-final adult height.

### 2.6 Software and reproducibility

Data management, descriptive analyses, and figure preparation were conducted in R 4.3.2. The model matrices, ordinary least-squares fits, 20 chained-equation imputations, pooled estimates, rank/VIF/condition-index checks, and residual diagnostics were independently verified in Python 3.12.13 using pandas, scikit-learn, and statsmodels. Reporting follows the Strengthening the Reporting of Observational Studies in Epidemiology (STROBE) recommendations for observational studies (33). Versioned R and Python scripts, a dependency manifest, output-to-figure/table map, and aggregate verification outputs are available at https://github.com/rongwei007/puberty. The clinical datasets are not publicly available because of patient privacy and institutional data-governance requirements.

## 3 Results

### 3.1 Cohort flow and NFAH ascertainment

Of the 235 children with CPP in the locked analytic cohort (125 GnRHa only and 110 GnRHa + rhGH), 91 (38.7%) had a measured 24-month height and 94 (40.0%) met at least one sex-appropriate NFAH criterion—53 in the GnRHa-only arm and 41 in the combined arm (**Figure 1**; **Table 3**). The criterion-confirmed NFAH set comprised 91 girls and 3 boys. Criteria B (≥2 years after menarche), C (height change < 1 cm over 6 months), and A (chronological-age or last-recorded-bone-age threshold) accounted for 35 (37.2%), 29 (30.9%), and 22 (23.4%) patients, respectively; criteria D and E accounted for 6 (6.4%) and 2 (2.1%). Mean age at NFAH ascertainment ranged from 13.9 to 15.2 years across criteria. The mean interval from baseline to NFAH was 5.19 years (SD 0.53; n = 93), whereas the mean last on-protocol height visit within the 0–24-month series was 18.6 months (SD 5.9). These are distinct intervals. The mean last-recorded bone age ranged from 13.0 to 14.5 years and was not measured contemporaneously with NFAH ascertainment (**Table 2**).

Because the NFAH definition allowed several maturity proxies, criterion-restriction analyses were performed. The primary female M2 estimate was +2.26 cm (95% CI 0.12 to 4.40; n = 91). Excluding the two criterion-E records gave +2.10 cm (−0.02 to 4.23; n = 89), and restricting the cohort to criteria A–C gave +1.73 cm (−0.49 to 3.94; n = 83). The direction was unchanged, but confidence intervals widened and included the null in the stricter definitions. Exact time since treatment discontinuation and last-year growth velocity were not uniformly encoded.

### 3.2 Baseline characteristics, the PAH imbalance, and IGF-1 monitoring

The treatment groups differed at baseline (**Table 1**). Absolute standardized mean differences exceeded 0.2 for age (1.21), bone age (1.13), sex composition (0.60), IGF-1 (0.38), baseline height (0.35), and BA–CA (0.23); BMI (0.16) and weight (0.11) were below 0.2 (**Figure 2**). In the criterion-confirmed NFAH set, mean chart-recorded PAH was 158.01 cm in the GnRHa-only arm and 159.68 cm in the combined arm. IGF-1 monitoring was strongly treatment-associated: baseline IGF-1 was available in 25/53 GnRHa-only records (47.2%) and 34/41 combined-therapy records (82.9%) in the criterion-confirmed all-sex set; the corresponding counts in the female primary cohort were 25/51 and 33/40. This pattern is clinically plausible because children receiving rhGH were monitored more intensively, but it means that IGF-1 missingness cannot be assumed to be unrelated to treatment. Chart-recorded PAH had a narrow overall distribution (SD 1.71 cm), was weakly correlated with baseline bone age, and was associated with treatment allocation; these features supported its diagnostic, rather than primary, role.

**Figure 2:**
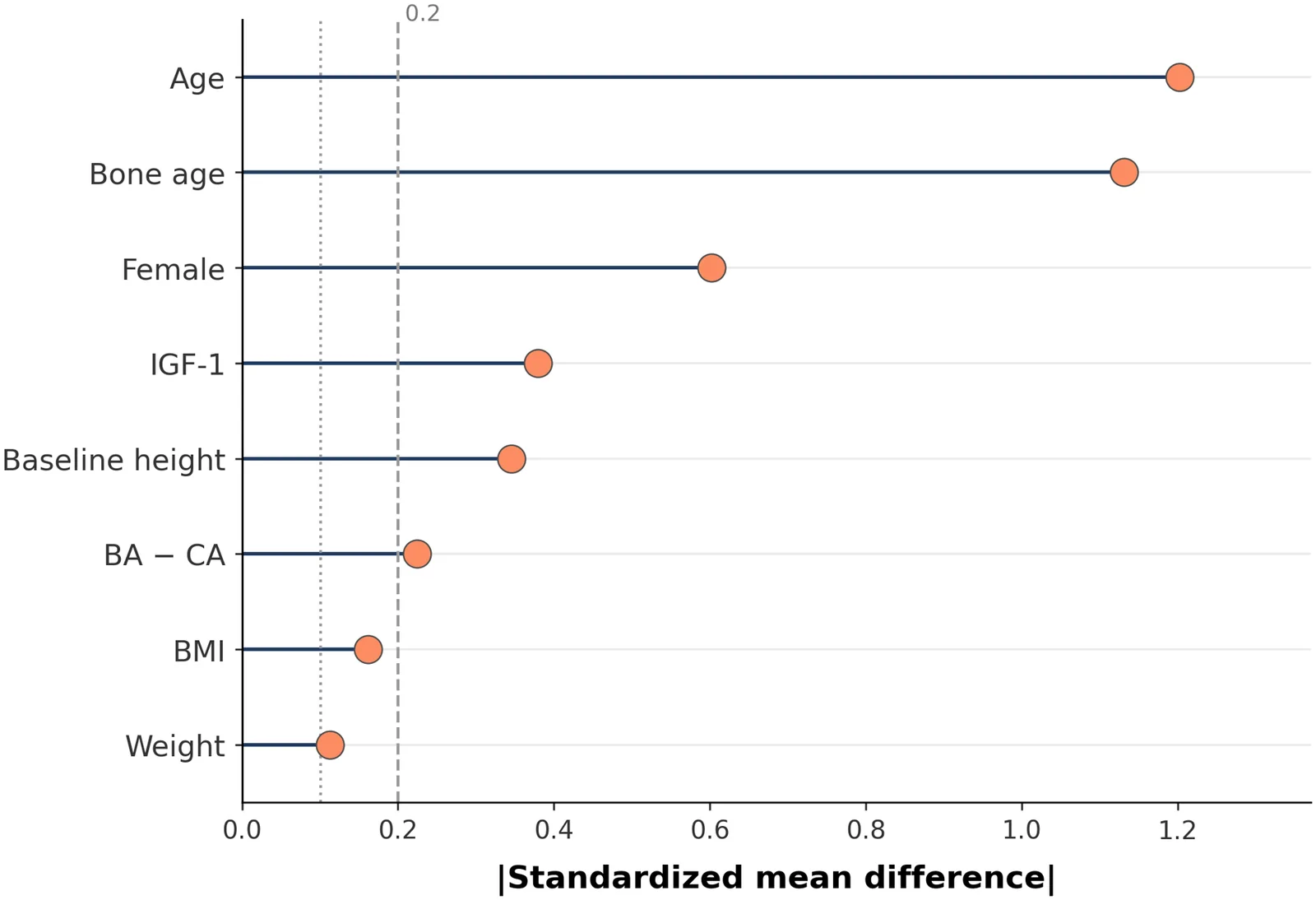
Baseline covariate balance between treatment arms, shown as absolute standardized mean differences for age, bone age, sex composition, IGF-1, baseline height, BA–CA, BMI, and weight in the locked cohort (N = 235). Dotted and dashed reference lines mark 0.1 and 0.2. Chart-recorded PAH is retained as a clinical-record field but was not selected for primary causal adjustment because its calculation method and timing were not uniformly documented.

### 3.3 Longitudinal growth dynamics and the 24-month endpoint

In girls with calculable height gain, mean gain was 13.22 cm (SD 2.22, n = 37) with GnRHa + rhGH and 11.00 cm (SD 1.96, n = 40) with GnRHa alone. The crude difference was +2.22 cm (95% CI 1.27 to 3.17). Sequential treatment estimates within the same sample were +2.22, +3.31, +3.31, +3.34, +3.34, +3.23, and +3.20 cm, respectively, for treatment only and after the successive addition of age, BMI, IGF-1, BA–CA, baseline height, and chart PAH. The primary M2 estimate was +3.23 cm (2.00 to 4.46; p < 0.001). In the parallel attained-height analysis, the estimates were +4.40 cm unadjusted, +0.48 cm in M1, + 3.24 cm in M2, and +3.17 cm in M3. Thus, the marked M1-to-M2 change occurred when baseline height was added; it did not arise from rank deficiency because all reported matrices were full rank. M1 adjusts for age and other determinants of attained height but omits the patient’s starting height, whereas M2 is the prespecified ANCOVA specification. Residual normality was poor for attained-height M1 (median Shapiro–Wilk p < 0.001 across imputations) and acceptable for M2 (median p = 0.605); median Breusch–Pagan p was 0.598 for M2. Change in height SDS gave concordant results: −0.24 (SD 0.32) with GnRHa alone and +0.20 (SD 0.36) with combined therapy, with an M2 estimate of +0.46 SDS (0.27 to 0.66; p < 0.001) (**Figure 3**; **Tables 4**, **5**).

**Table 4:** Longitudinal growth dynamics by treatment group.

Metric	GnRHa only	GnRHa + rhGH
Baseline height (all-sex locked cohort, cm)	136.98 (6.57) [n = 124]	139.33 (7.04) [n = 109]
Height at 24 months (all-sex locked cohort, cm)	144.63 (4.59) [n = 41]	151.66 (7.43) [n = 50]
Height gain 0–24 months (all-sex locked cohort, cm)	11.00 (1.96) [n = 40]	13.50 (2.23) [n = 50]
Near-final adult height (criterion-confirmed all-sex, cm)	160.10 (3.79) [n = 53]	161.81 (3.88) [n = 41]
Chart-recorded baseline PAH (criterion-confirmed all-sex, cm)	158.01 (1.46) [n = 53]	159.68 (1.55) [n = 41]
Recalculated BP-PAH (criterion-confirmed all-sex calculable subset, cm)	148.06 (6.67) [n = 36]	143.51 (3.79) [n = 23]
NFAH minus chart-recorded baseline PAH (criterion-confirmed all-sex, cm)	2.09 (3.56) [n = 53]	2.14 (3.55) [n = 41]
Baseline height SDS (girls, 2009 Chinese reference)	0.82 (0.98) [n = 121]	−0.07 (0.82) [n = 84]
Height SDS at 24 months (girls)	0.56 (0.91) [n = 41]	−0.02 (0.67) [n = 37]
Δ height SDS 0–24 months (girls)	−0.24 (0.32) [n = 40]	0.20 (0.36) [n = 37]

Values are mean (standard deviation) [available n]. Rows explicitly identify the analysis population: the first three rows are all-sex locked-cohort summaries; the next five rows are all-sex criterion-confirmed NFAH summaries; and the final three rows are girls-only summaries. Baseline corresponds to month 0; 24 months is the on-treatment secondary endpoint; NFAH is near-final adult height. Recalculated BP-PAH is shown for the calculable subset as a descriptive method comparison. Height SDS was computed with the LMS method using the girls’ height-for-age parameters of the 2009 Chinese reference (29); SDS at 24 months uses baseline decimal age plus 2 years.

**Figure 3:**
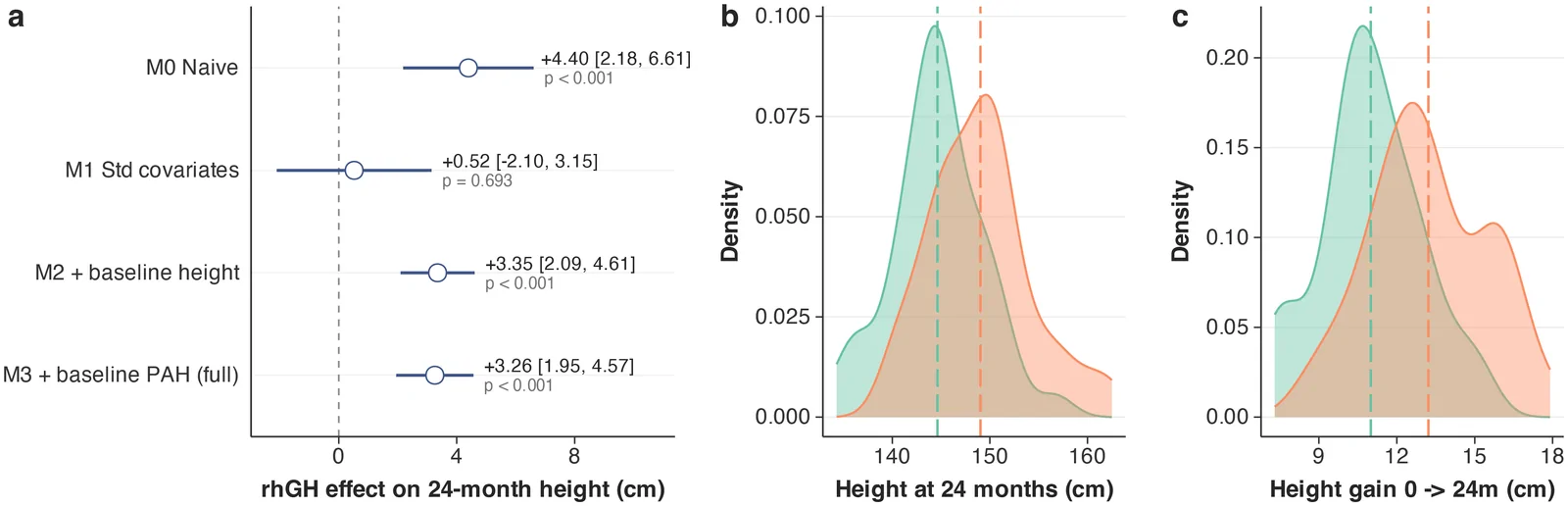
Twenty-four-month on-treatment growth. **(a)** Pooled treatment estimates after 20 chained-equation imputations: M0 treatment only; M1 plus baseline age, BMI, IGF-1, and BA–CA; M2 plus baseline height; M3 plus chart-recorded PAH; and ANCOVA of attained 24-month height with baseline height included. Lines are 95% confidence intervals. **(b)** Height-gain distributions by treatment group. **(c)** Change in height-SDS distributions in girls.

**Table 5:** Full-rank treatment-effect models for the 24-month and near-final adult height endpoints.

Endpoint	Model	n	Estimate [95% CI]	p-value
Height gain 0–24 months (cm)	M0	77	+2.22 [1.27, 3.17]	< 0.001
Height gain 0–24 months (cm)	M1	77	+3.34 [2.16, 4.52]	< 0.001
Height gain 0–24 months (cm)	Primary M2	77	+3.23 [2.00, 4.46]	< 0.001
Height gain 0–24 months (cm)	Diagnostic M3	77	+3.20 [1.93, 4.47]	< 0.001
Attained height at 24 months (cm)	M0	78	+4.40 [2.18, 6.61]	< 0.001
Attained height at 24 months (cm)	M1	78	+0.48 [−2.19, 3.16]	0.719
Attained height at 24 months (cm)	Primary M2 (ANCOVA)	78	+3.24 [2.03, 4.46]	< 0.001
Attained height at 24 months (cm)	Diagnostic M3	78	+3.17 [1.90, 4.43]	< 0.001
Δ height SDS 0–24 months	M0	77	+0.44 [0.28, 0.59]	< 0.001
Δ height SDS 0–24 months	Primary M2	77	+0.46 [0.27, 0.66]	< 0.001
Near-final adult height (cm)	M0	91	+1.70 [0.08, 3.32]	0.040
Near-final adult height (cm)	M1	91	+2.21 [0.19, 4.23]	0.032
Near-final adult height (cm)	Primary M2	91	+2.26 [0.12, 4.40]	0.039
Near-final adult height (cm)	Diagnostic M3	91	+0.58 [−1.60, 2.75]	0.598
Bone age at 24 months (years)	M0	83	+0.68 [0.27, 1.09]	0.001
Bone age at 24 months (years)	Primary M2	83	+0.00 [−0.27, 0.27]	0.997
Bone-age progression 0–24 months (years)	M0	63	−0.19 [−0.32, −0.06]	0.004
Bone-age progression 0–24 months (years)	Primary M2	63	−0.14 [−0.30, 0.03]	0.097

Effect estimates are mean differences with 95% confidence intervals; positive values favor GnRHa + rhGH. Height, height-gain, height-SDS, and NFAH models use the female-only outcome-specific samples and 20 chained-equation imputations for missing baseline covariates. M0 contains treatment only; M1 adds baseline age, BMI, IGF-1, and BA–CA; M2 adds baseline height and is the primary adjusted model; M3 adds chart-recorded PAH and is diagnostic. Baseline bone age is not separately entered. ANCOVA refers to attained 24-month height with baseline height included. The bone-age rows use the separate criterion-confirmed female NFAH cohort: n = 83 for bone age at 24 months and n = 63 for progression among girls with both readings. These are not subsets of the n = 78/77 height-outcome cohorts. Full coefficients and diagnostics are in **Supplementary Table S1–S2**.

### 3.4 Near-final adult height and PAH sensitivity analysis

Among the 91 girls in the primary NFAH cohort, mean NFAH was 160.17 cm (SD 3.83, n = 51) with GnRHa alone and 161.87 cm (SD 3.91, n = 40) with GnRHa + rhGH. The unadjusted association was +1.70 cm (95% CI 0.08 to 3.32; p = 0.040). The full-rank M1 estimate was +2.21 cm (0.19 to 4.23), and the primary PAH-free M2 estimate was +2.26 cm (0.12 to 4.40; p = 0.039). Adding chart-recorded PAH in diagnostic M3 attenuated the estimate to +0.58 cm (−1.60 to 2.75; p = 0.598) (**Figure 4**; **Table 5**). Because chart PAH may partly record treatment-selection intent, the M3 change is informative about sensitivity to PAH conditioning but is not interpreted as a definitive causal correction.

**Figure 4:**
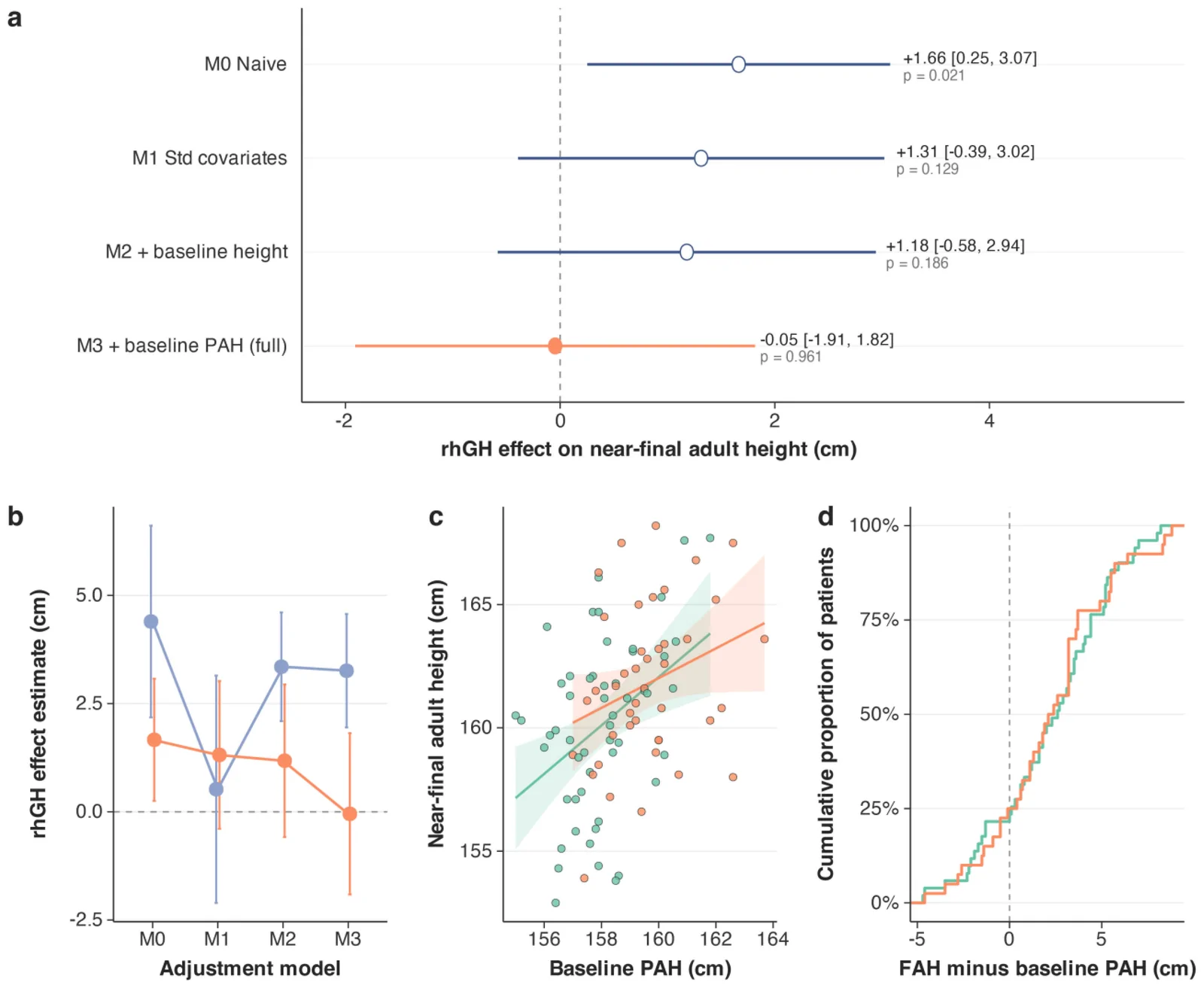
Near-final adult height analyses. **(a)** Full-rank treatment estimates from M0–M3 in the female NFAH cohort (n = 91); M2 is the primary PAH-free adjusted model, and M3 is diagnostic. **(b)** Treatment estimates for 24-month height gain and NFAH across the same covariate sequence. **(c)** NFAH versus chart-recorded baseline PAH. **(d)** Empirical distributions of NFAH minus chart-recorded PAH by treatment arm.

The complete coefficient tables and diagnostics showed no automatic term omission in the NFAH models. For M2, the median condition index was 3.14, median maximum VIF was 2.66, median Breusch–Pagan p was 0.839, median Shapiro–Wilk p was 0.764, and median maximum Cook’s distance was 0.117 across the 20 imputations. Chart-recorded PAH was positively correlated with NFAH (r = 0.42) but had a narrow range; recalculated BP-PAH was more dispersed but underestimated NFAH in its calculable subset. These observations concern the use of PAH as an adjustment covariate and do not alter the case-level clinical measurements used for the NFAH and 24-month outcomes.

### 3.5 Achievement versus prediction and the joint trajectory

In the female NFAH cohort, mean NFAH minus chart-recorded baseline PAH was 2.13 cm (SD 3.58) with GnRHa alone and 2.17 cm (SD 3.59) with combined therapy (**Figure 5**). Individual trajectories from baseline through the 24-month visit to NFAH and the corresponding group means are shown descriptively in **Figure 6**. The combined arm showed a larger separation at 24 months than at NFAH, consistent with the distinction between an on-treatment growth response and an observational near-adult-height association.

**Figure 5:**
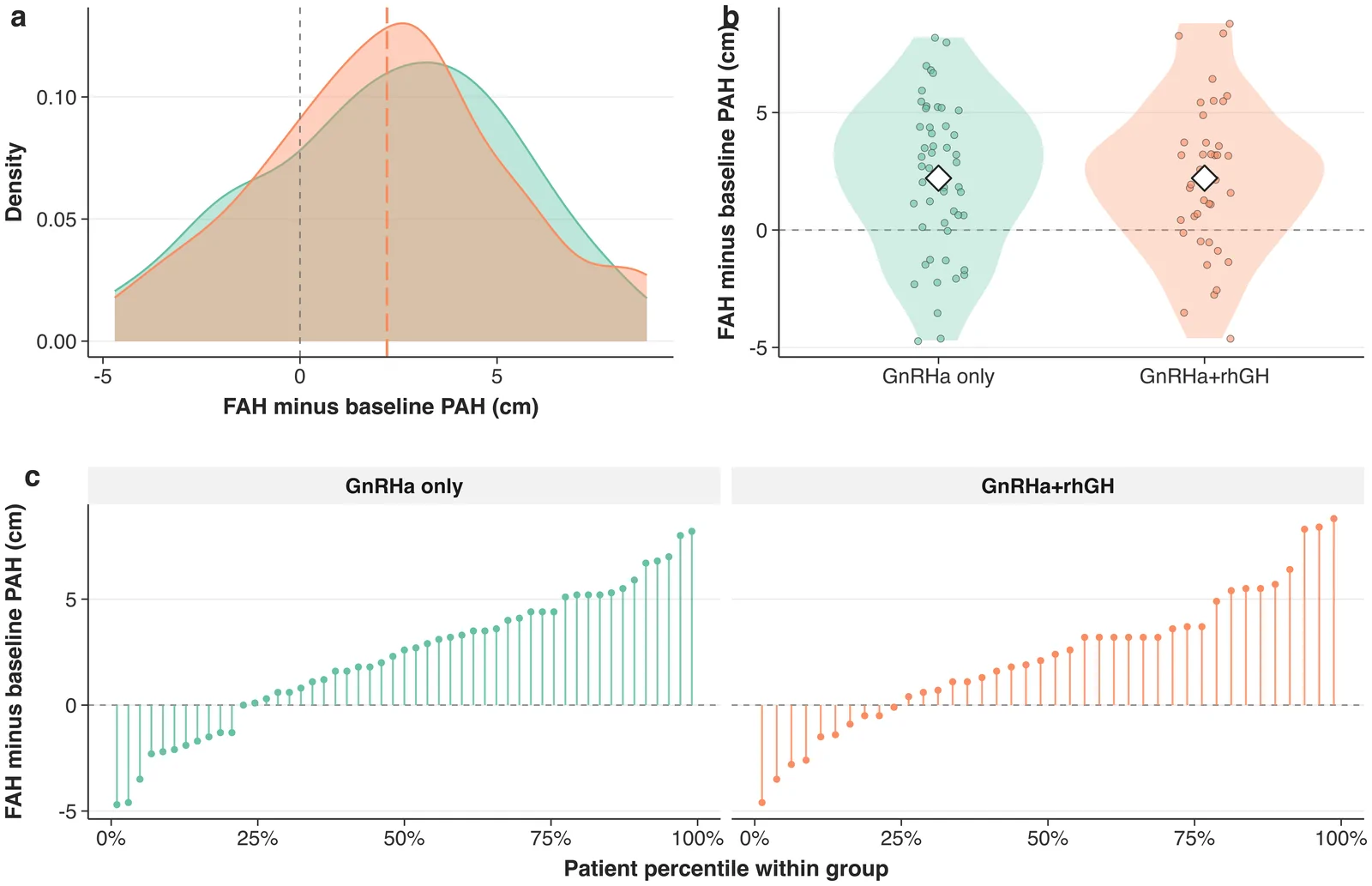
NFAH minus chart-recorded baseline PAH in the female NFAH cohort (n = 91; 51 GnRHa only and 40 GnRHa + rhGH). **(a)** Density by treatment group; the vertical dashed line marks zero, and long-dashed lines mark group means. **(b)** Violin and individual observations; diamonds mark group means. **(c)** Patient-level values sorted within treatment arm. This is a descriptive achievement-versus-chart-prediction display.

**Figure 6:**
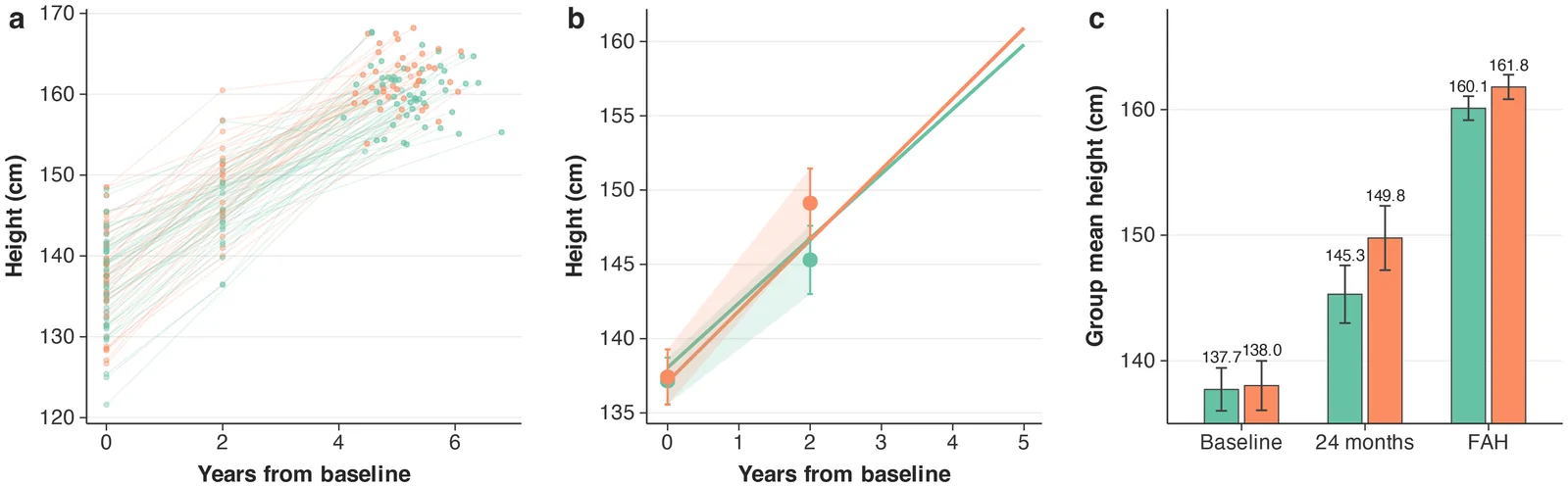
Descriptive joint height trajectories. **(a)** Patient-level height records from baseline through 24 months and NFAH in the female NFAH cohort. **(b)** Group-level fitted trajectories with 95% confidence bands. **(c)** Group means with 95% confidence intervals at baseline, 24 months, and NFAH.

### 3.6 Complete-case and criterion-restriction sensitivity analyses

For NFAH M2, a complete-case analysis including IGF-1 gave +3.70 cm (95% CI 0.50 to 6.89; n = 45), and a complete-case analysis omitting IGF-1 to increase sample size gave +4.34 cm (1.80 to 6.88; n = 60), compared with the pooled 20-imputation estimate of +2.26 cm (0.12 to 4.40; n = 91). The complete-case point estimates were therefore 1.44–2.08 cm larger than the imputed estimate; this is evidence of systematic sample/monitoring differences, not merely loss of precision. For 24-month height gain, complete-case M2 estimates were +2.44 cm (0.69 to 4.19; n = 36) with IGF-1 and +3.43 cm (2.07 to 4.79; n = 53) without IGF-1, compared with the pooled estimate of +3.23 cm. Direction was preserved, but the magnitude differences reinforce cautious interpretation of models containing a treatment-associated monitoring variable. Criterion-restricted NFAH estimates are reported in Section 3.1.

### 3.7 Bone-age sensitivity endpoint and exploratory heterogeneity

The bone-age sensitivity analyses were conducted in the criterion-confirmed female NFAH cohort using the supplementary chart abstraction, not in the 78/77-girl 24-month height cohorts. Among 83 girls with a 24-month bone-age reading, the crude association with combined therapy was +0.68 years (95% CI 0.27 to 1.09) and the M2 estimate was +0.00 years (−0.27 to 0.27). Among 63 girls with both baseline and 24-month bone age, progression was −0.19 years in the crude analysis (−0.32 to −0.06; p = 0.004) and −0.14 years in M2 (−0.30 to 0.03) (**Figure 7**; **Table 5**). Adjunctive rhGH was therefore not associated with accelerated skeletal maturation under ongoing GnRHa suppression. PAH-stratified and causal-forest results are shown in **Figure 8** solely as exploratory descriptions; the small cohort and the diagnostic status of chart PAH preclude confirmatory subgroup interpretation. The exploratory conditional average treatment effect (CATE) distribution was centered at approximately +0.8 cm, below the pooled M2 estimate of +2.26 cm; quartile-specific estimates were +0.45, +1.49, +1.91, and −0.66 cm. This pattern is consistent with the imprecision and model sensitivity of the adult-height association and was not used to revise the primary estimate.

**Figure 7:**
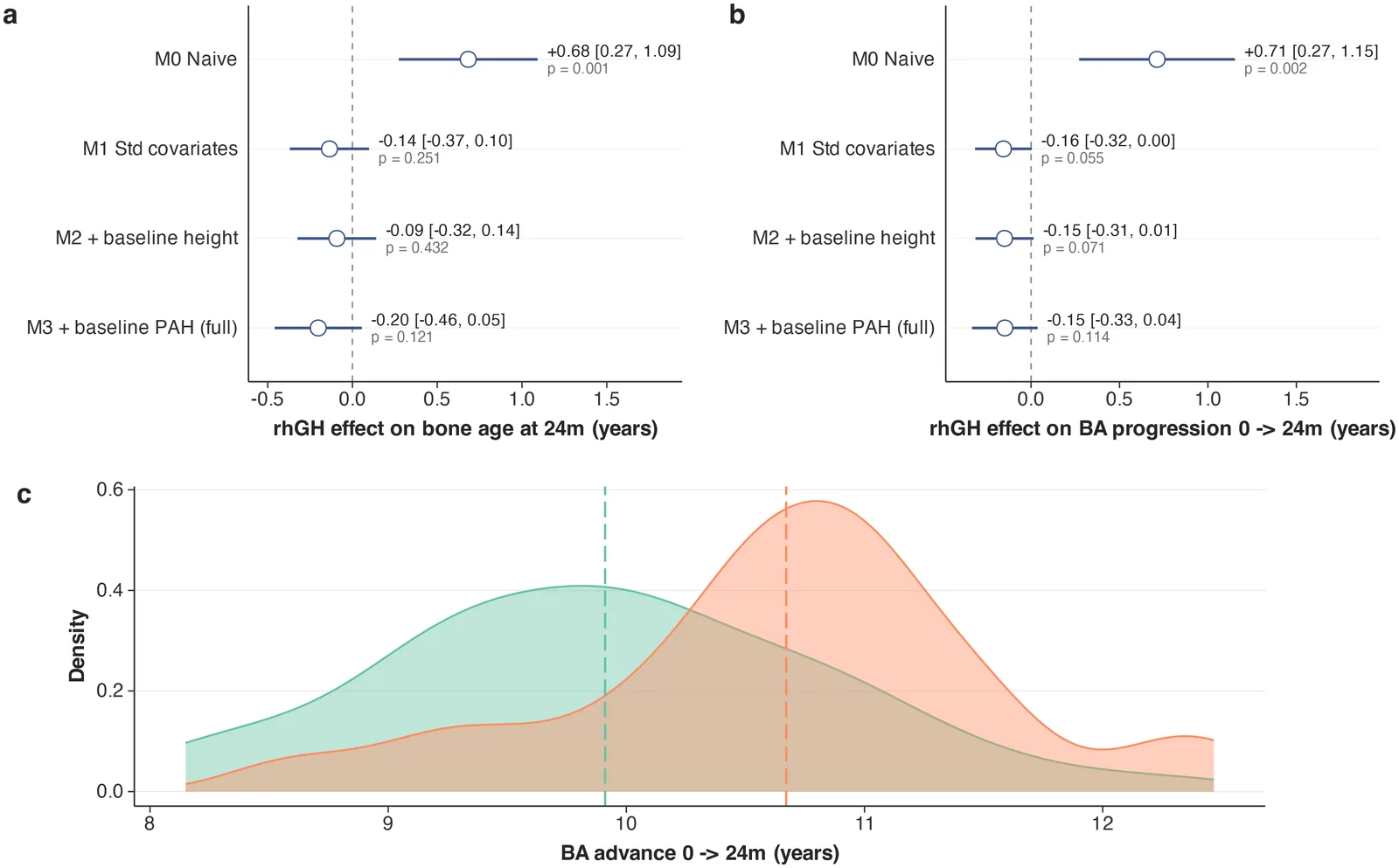
Bone-age sensitivity analyses. **(a)** Covariate cascade for bone age at 24 months in the criterion-confirmed female NFAH cohort (n = 83). **(b)** Cascade for bone-age progression among girls with both baseline and 24-month readings (n = 63). **(c)** Unadjusted all-sex descriptive distribution of the 0–24-month bone-age increment among all 66 locked-cohort patients with both readings; the three additional observations are from boys, which explains the difference from panel **(b)**.

**Figure 8:**
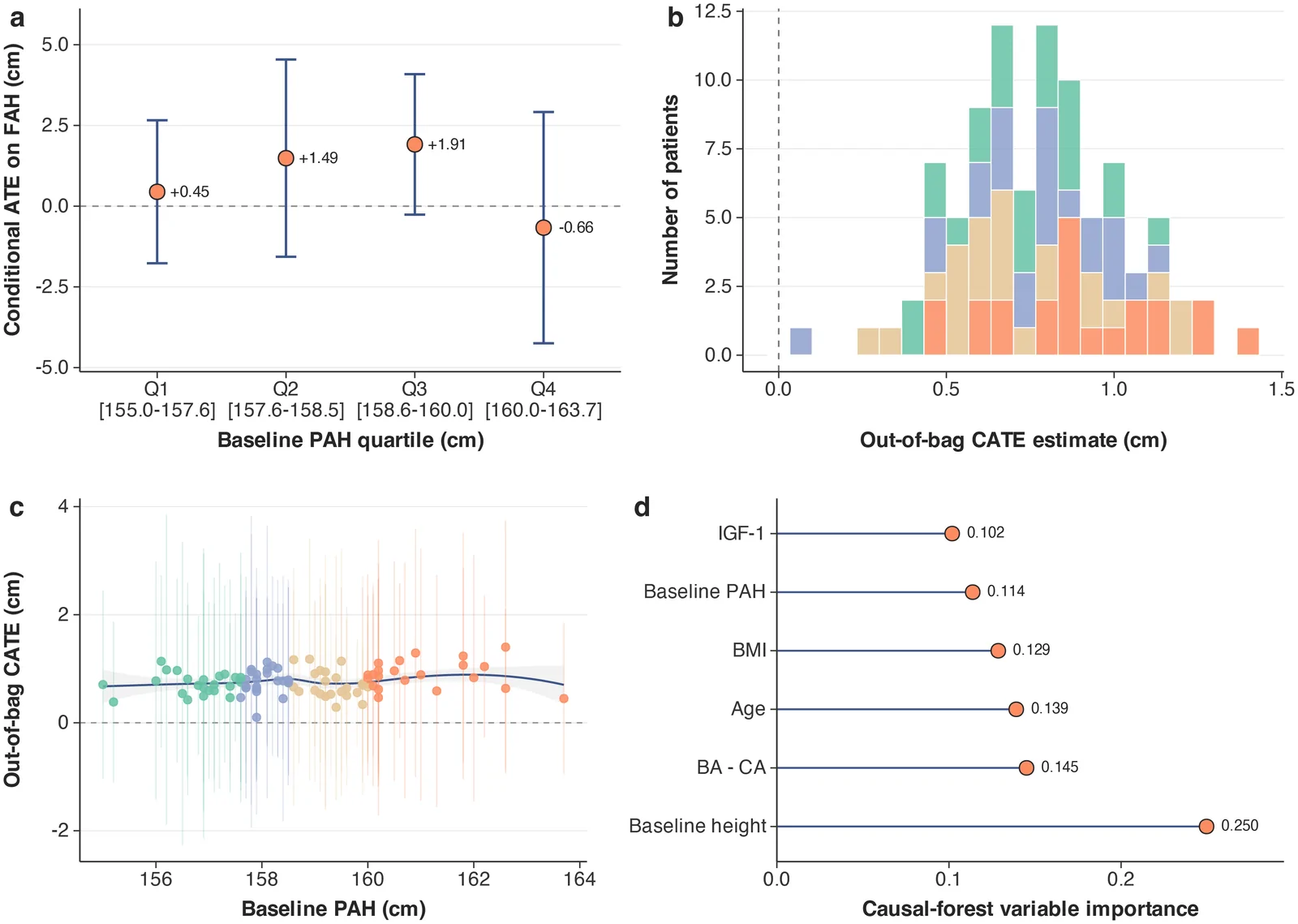
Exploratory heterogeneous-treatment-effect displays in the female NFAH cohort. PAH-stratified and causal-forest estimates (32) are descriptive only because of the small cohort and the diagnostic role of chart-recorded PAH; they were not used for the primary conclusion.

## 4 Discussion

In this single-center cohort of children with CPP, adjunctive rhGH was associated with higher on-treatment growth and a positive but imprecisely estimated NFAH difference. The primary PAH-free M2 association with NFAH was +2.26 cm (95% CI 0.12 to 4.40), whereas the diagnostic chart-PAH-conditioned estimate was +0.58 cm (−1.60 to 2.75). Stricter ascertainment definitions retained the positive direction, but their confidence intervals included the null. At 24 months, height gain, ANCOVA of attained height, and change in height SDS all showed concordant associations, without accelerated bone-age progression. These results support a short-term growth association; the adult-height magnitude remains uncertain because of observational treatment selection, modest sample size, heterogeneous NFAH criteria, and sensitivity to PAH adjustment.

Prior meta-analyses (18, 19, 23, 24) emphasized 12–24-month gain and rarely reported the provenance, distribution, or calibration of PAH variables. The present analysis shows why a chart-recorded PAH field should not be treated as a routine adjustment variable without documenting its formula, timing, physiological correlations, and common support. In clinical records, PAH may reflect prognosis, clinician judgment, and treatment-selection intent simultaneously.

Predicted adult height remains clinically relevant, and all PAH values used here were recorded in or calculated from the clinical records. The concern is narrower: the chart field did not retain a uniform formula and timestamp for retrospective causal adjustment, and the recalculated BP-PAH showed limited calibration in the available subset (**Figure 9**). This caution is limited to covariate interpretation and does not change the status of the recorded clinical measurements or the Bayley–Pinneau method itself.

**Figure 9:**
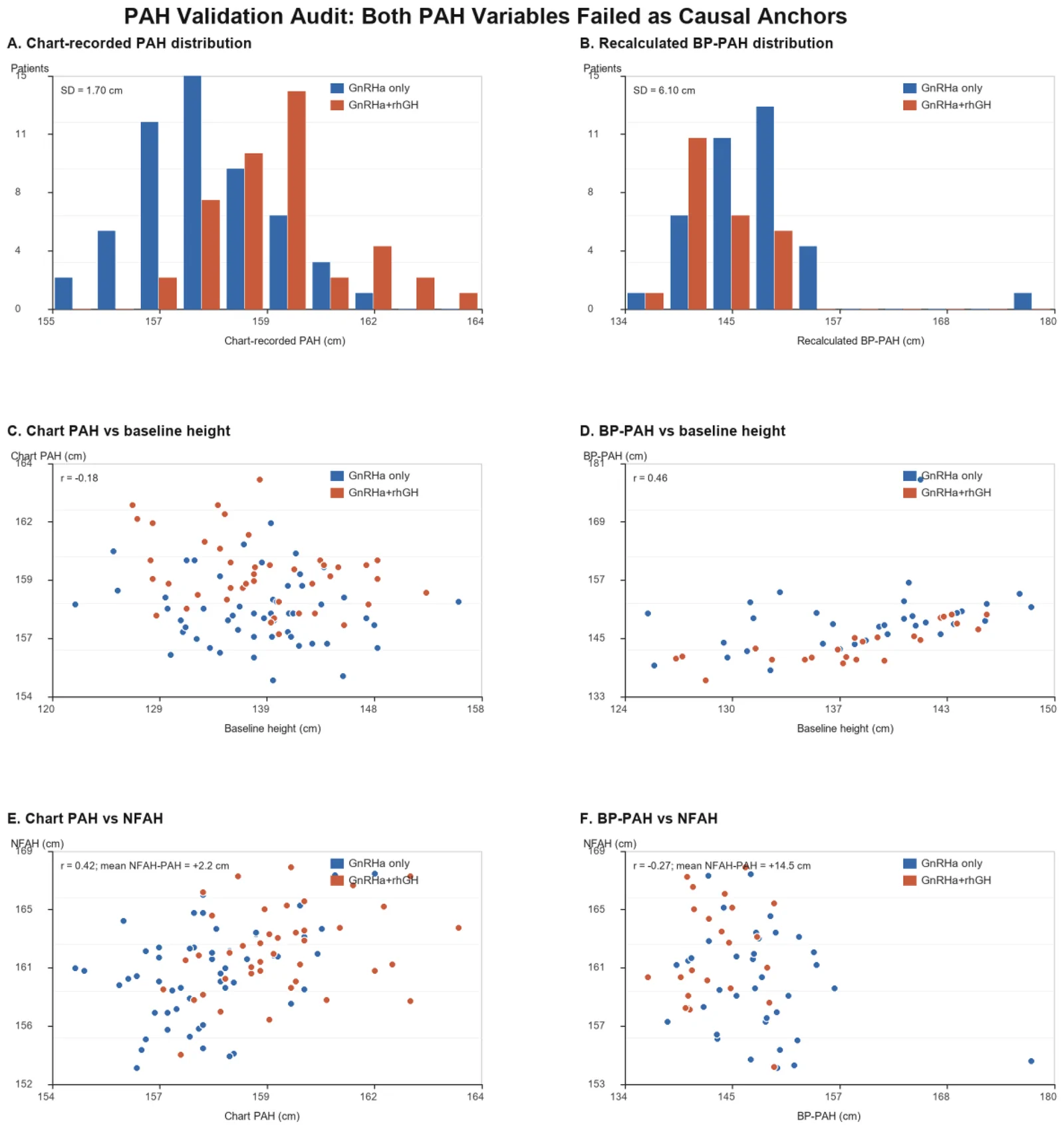
PAH method-comparison audit. Panels **(A–F)** show the distributions of chart-recorded PAH and recalculated BP-PAH, their relationships with baseline height, and their relationships with NFAH. Chart-recorded PAH was retained as a diagnostic covariate, whereas recalculated BP-PAH was used only as a descriptive method comparison; neither was used as the primary causal adjustment variable.

The 24-month fluctuation is explained by specification. M1 adjusts attained height for age and other baseline features while omitting baseline height; M2 adds baseline height and therefore becomes a conventional ANCOVA. The treatment estimate consequently changed from +0.48 cm in M1 to +3.24 cm in M2. By contrast, the height-gain and height-SDS outcomes were stable across the same sequence. Full-rank matrices, modest VIFs and condition indices, and acceptable M2 residual diagnostics make deterministic collinearity an unlikely explanation for the observed coefficient fluctuation.

The exploratory heterogeneous-treatment-effect displays suggested substantial individual variation but did not identify a stable subgroup for clinical decision-making. These analyses were not used to modify the primary estimate or the study conclusion.

The model audit improves interpretability by separating the standard M1 covariate set from baseline height, removing the deterministic age/bone-age/BA–CA dependency, reporting the exact formulas, and demonstrating that all reported matrices are full rank with no automatically omitted variables. For NFAH, adding baseline height contributed almost no incremental fit: its pooled coefficient in M2 was 0.0147 cm per baseline cm (p = 0.887), median R² was 0.101 in both M1 and M2, median adjusted R² decreased from 0.048 to 0.037, and median Akaike information criterion (AIC) increased from 508.9 to 510.9. M2 remains the primary model because baseline height was clinically prespecified, not because it outperformed M1. The weak coefficient may reflect the restricted NFAH range (SD 3.8–3.9 cm) within a maturity-ascertained cohort relative to the wider baseline-height range and residual measurement and ascertainment heterogeneity.

Several limitations must be acknowledged. First, this was a single-center retrospective study with a modest female NFAH cohort, limiting precision and overlap. Second, pubertal phenotype details, treatment adherence, interruptions, exact discontinuation dates, and some baseline covariates were not uniformly encoded. Third, IGF-1 availability was strongly treatment-associated, and complete-case NFAH estimates were materially larger than the multiply imputed estimate; chained-equation imputation cannot guarantee removal of bias when missingness depends on unrecorded monitoring decisions. Fourth, NFAH was ascertained through several criteria and should not be treated as strict final adult height in every patient. Fifth, bone age was not generally obtained on the NFAH ascertainment date. Sixth, chart PAH was populated only in the NFAH-focused abstraction records, and its formula and timing were not uniform enough for it to serve as the primary causal adjustment variable. Finally, treatment selection was observational and may remain confounded by clinician judgment, family preference, growth expectations, and unrecorded adherence; no statistical adjustment can be assumed to remove these factors completely.

The apparently low mean bone age in criterion A is explained by two features. Criterion A could be met through either the chronological-age threshold or the last-recorded-bone-age threshold, so not every patient was required to exceed the bone-age cutoff. In addition, the **Table 2** bone-age columns refer to baseline, 24 months, and the last recorded reading, not to a radiograph obtained on the NFAH ascertainment date. Residual growth therefore cannot be excluded in every criterion-A patient, which is why the endpoint is termed NFAH and criterion-restricted analyses are reported.

Three priorities follow. First, future CPP studies should preserve PAH field provenance, formula, timing relative to treatment decisions, source table, maturity-category choice, and raw height/bone-age inputs. Second, PAH must be validated against NFAH or FAH within the study population before it is used for stratification or causal adjustment. Third, prospective studies should report both short-term growth response and adult-height outcomes while avoiding strong adult-height claims from PAH-conditioned retrospective models unless the PAH variable passes validation.

## 5 Conclusion

In this single-center retrospective CPP cohort, adjunctive rhGH was associated with greater 24-month growth: +3.23 cm in the primary height-gain model (95% CI, 2.00 to 4.46), +3.24 cm in ANCOVA of attained height (2.03 to 4.46), and +0.46 SDS (0.27 to 0.66), without accelerated bone-age progression. The primary PAH-free NFAH association was +2.26 cm (0.12 to 4.40), but the estimate was attenuated after chart-PAH conditioning and became less precise under stricter NFAH criteria. The findings support an on-treatment growth advantage and a possible NFAH association, but they do not establish a causal or definitive adult-height benefit.

## Data Availability

The raw data supporting the conclusions of this article will be made available by the authors, without undue reservation.
