# Supplementary material for: Near-final adult height after GnRHa plus growth hormone in central precocious puberty: a single-center cohort study and predicted adult height validation audit
Source: Front Endocrinol (Lausanne). 2026 Sep 18;17:1885575. doi: 10.3389/fendo.2026.1885575 (PMC13630612; doi:10.3389/fendo.2026.1885575)
Supplement: Supplementary file 1 [file SupplementaryFile1.docx]

# Supplementary Figures

Figure S1. Growth-velocity dynamics by treatment group. (a) Violin plus boxplot of growth velocity over the 0-6, 6-12, and 12-24 month windows; both arms are shown side-by-side within each window. (b) Group-mean velocity bars with 95% confidence intervals for the same three windows; numeric labels indicate the group mean (cm/year).

Figure S2. Attrition profile and inverse-probability-of-censoring weights for the 24-month observation model. (a) Retention curve showing the percentage of patients with an observed height at each canonical month from baseline to 24 months, by treatment group; dotted line marks 50% retention. (b) Density of stabilized inverse-probability-of-censoring weights on the log10 scale; vertical dashed line marks weight = 1.

Figure S3. IGF-1 monitoring intensity and group-mean trajectory. (a) Histogram of per-patient IGF-1 measurement counts across the 0-39 month follow-up window by treatment group. (b) Group-mean IGF-1 trajectory from baseline to 24 months with 95% confidence-interval ribbons.

Figure S4. Sex composition and full-cohort cascade sensitivity. (a) Sex composition by treatment arm shown as a 100% stacked bar; female-only primary analysis is justified by the marked sex imbalance. (b) Side-by-side cascade comparison of the female-only primary cohort (n = 91, orange) and the full NFAH-evaluable cohort (n = 95 including 4 males, navy); points are point estimates with 95% CI error bars.

Figure S5. MICE m = 5 multiple-imputation sensitivity analysis on the full NFAH cascade. Side-by-side comparison of the primary median-imputation pipeline (navy) and a multiple-imputation pipeline using chained equations with m = 5 (orange). Point estimates, 95% CIs, and the MICE-pooled M3 estimate are shown.

Figure S6. Best linear projection of the causal-forest conditional average treatment effect (CATE). Coefficients (with 95% CIs and p values) of the best linear projection of the per-patient CATE on the six baseline covariates used in the M3 cascade. No covariate-driven HTE gradient reached statistical significance.
