# Supplementary material for: Near-final adult height after GnRHa plus growth hormone in central precocious puberty: a single-center cohort study and predicted adult height validation audit
Source: Front Endocrinol (Lausanne). 2026 Sep 18;17:1885575. doi: 10.3389/fendo.2026.1885575 (PMC13630612; doi:10.3389/fendo.2026.1885575)
Supplement: Supplementary file 2 [file SupplementaryFile3.docx]

Supplementary Tables S1–S2: Full-Rank Models and Diagnostics

*Companion to Near-Final Adult Height After GnRHa Plus Growth Hormone in Central Precocious Puberty*

All models are ordinary least-squares linear models in female-only analytic cohorts. M0 includes treatment only; M1 adds baseline age, BMI, IGF-1 and BA–CA; M2 adds baseline height and is the primary adjusted model; M3 adds chart-recorded baseline PAH and is diagnostic. Baseline bone age is not entered separately because bone age = chronological age + BA–CA. Sex is constant in these cohorts and is omitted.

Missing baseline covariates were handled with 20 chained-equation imputations using posterior draws; observed outcomes were not imputed. Coefficients and standard errors are pooled with Rubin's rules. Model-fit and residual statistics are summarized as the median [minimum, maximum] across imputations because those statistics do not have a standard Rubin pooling rule. VIFs listed by term are from the first imputation; their maximum across all terms is also summarized over all imputations. No patient record was deleted on the basis of a residual or influence measure.

# Table S1. Complete model specification and diagnostics

## S1a. Model formulas, design-matrix rank and collinearity

| **Endpoint** | **Model** | **Formula** | **n** | **Columns** | **Rank(s)** | **Automatically omitted** | **Standardized condition index** | **Maximum VIF** |
| --- | --- | --- | --- | --- | --- | --- | --- | --- |
| Near-final adult height (cm) | M0 | treatment | 91 | 2 | 2 | None | 1.00 [1.00, 1.00] | 1.00 [1.00, 1.00] |
| Near-final adult height (cm) | M1 | treatment + age + BMI + IGF-1 + BA–CA | 91 | 6 | 6 | None | 2.27 [1.97, 2.52] | 1.69 [1.49, 1.82] |
| Near-final adult height (cm) | M2 | M1 + baseline height | 91 | 7 | 7 | None | 3.14 [2.87, 3.39] | 2.66 [2.28, 2.89] |
| Near-final adult height (cm) | M3 | M2 + chart-recorded PAH | 91 | 8 | 8 | None | 3.17 [2.93, 3.41] | 2.66 [2.28, 2.89] |
| Height gain, baseline to 24 months (cm) | M0 | treatment | 77 | 2 | 2 | None | 1.00 [1.00, 1.00] | 1.00 [1.00, 1.00] |
| Height gain, baseline to 24 months (cm) | M1 | treatment + age + BMI + IGF-1 + BA–CA | 77 | 6 | 6 | None | 2.57 [2.38, 2.75] | 1.99 [1.87, 2.20] |
| Height gain, baseline to 24 months (cm) | M2 | M1 + baseline height | 77 | 7 | 7 | None | 3.39 [3.24, 3.65] | 3.16 [2.93, 3.54] |
| Height gain, baseline to 24 months (cm) | M3 | M2 + chart-recorded PAH | 77 | 8 | 8 | None | 3.49 [3.26, 3.67] | 3.16 [2.98, 3.54] |
| Attained height at 24 months (cm) | M0 | treatment | 78 | 2 | 2 | None | 1.00 [1.00, 1.00] | 1.00 [1.00, 1.00] |
| Attained height at 24 months (cm) | M1 | treatment + age + BMI + IGF-1 + BA–CA | 78 | 6 | 6 | None | 2.63 [2.47, 2.74] | 2.06 [1.92, 2.17] |
| Attained height at 24 months (cm) | M2 | M1 + baseline height | 78 | 7 | 7 | None | 3.42 [3.24, 3.79] | 3.21 [2.90, 3.53] |
| Attained height at 24 months (cm) | M3 | M2 + chart-recorded PAH | 78 | 8 | 8 | None | 3.47 [3.24, 3.80] | 3.24 [2.90, 3.53] |
| Change in height SDS, baseline to 24 months | M0 | treatment | 77 | 2 | 2 | None | 1.00 [1.00, 1.00] | 1.00 [1.00, 1.00] |
| Change in height SDS, baseline to 24 months | M1 | treatment + age + BMI + IGF-1 + BA–CA | 77 | 6 | 6 | None | 2.60 [2.39, 2.77] | 2.01 [1.89, 2.25] |
| Change in height SDS, baseline to 24 months | M2 | M1 + baseline height | 77 | 7 | 7 | None | 3.39 [3.24, 3.64] | 3.15 [2.93, 3.54] |
| Change in height SDS, baseline to 24 months | M3 | M2 + chart-recorded PAH | 77 | 8 | 8 | None | 3.47 [3.25, 3.67] | 3.16 [2.97, 3.54] |
| Attained height audit | Former candidate matrix | treatment + age + BMI + IGF-1 + bone age + BA–CA | 78 | 7 | 6 | Bone age (aliased under recorded term order) | Not finite (exact dependence) | Undefined |

The former candidate matrix has the exact identity BA–CA = bone age − chronological age and therefore rank 6 for 7 columns. The corrected models omit bone age as a separate term by design; every corrected matrix was full rank in all 20 imputations and no software-driven omission occurred.

## S1b. Treatment estimates and model fit

| **Endpoint** | **Model** | **n** | **Treatment estimate** | **SE** | **95% CI** | **p** | **Median R²** | **Median adjusted R²** | **Median residual SE** | **Median AIC** |
| --- | --- | --- | --- | --- | --- | --- | --- | --- | --- | --- |
| Near-final adult height (cm) | M0: treatment only | 91 | 1.701 | 0.816 | [0.079, 3.323] | 0.040 | 0.047 | 0.036 | 3.865 | 506.3 |
| Near-final adult height (cm) | M1: standard covariates | 91 | 2.210 | 1.013 | [0.194, 4.226] | 0.032 | 0.101 | 0.048 | 3.840 | 508.9 |
| Near-final adult height (cm) | M2: + baseline height (primary) | 91 | 2.259 | 1.074 | [0.121, 4.398] | 0.039 | 0.101 | 0.037 | 3.862 | 510.9 |
| Near-final adult height (cm) | M3: + chart PAH (diagnostic) | 91 | 0.579 | 1.092 | [-1.595, 2.753] | 0.598 | 0.237 | 0.173 | 3.580 | 498.0 |
| Height gain, baseline to 24 months (cm) | M0: treatment only | 77 | 2.219 | 0.477 | [1.269, 3.168] | <0.001 | 0.224 | 0.214 | 2.090 | 334.0 |
| Height gain, baseline to 24 months (cm) | M1: standard covariates | 77 | 3.343 | 0.591 | [2.164, 4.522] | <0.001 | 0.391 | 0.349 | 1.902 | 323.3 |
| Height gain, baseline to 24 months (cm) | M2: + baseline height (primary) | 77 | 3.227 | 0.617 | [1.995, 4.459] | <0.001 | 0.395 | 0.343 | 1.910 | 324.8 |
| Height gain, baseline to 24 months (cm) | M3: + chart PAH (diagnostic) | 77 | 3.199 | 0.635 | [1.931, 4.468] | <0.001 | 0.397 | 0.335 | 1.921 | 326.6 |
| Attained height at 24 months (cm) | M0: treatment only | 78 | 4.395 | 1.111 | [2.182, 6.609] | <0.001 | 0.171 | 0.160 | 4.901 | 471.3 |
| Attained height at 24 months (cm) | M1: standard covariates | 78 | 0.483 | 1.339 | [-2.189, 3.156] | 0.719 | 0.386 | 0.344 | 4.331 | 455.8 |
| Attained height at 24 months (cm) | M2: + baseline height (primary) | 78 | 3.244 | 0.610 | [2.025, 4.462] | <0.001 | 0.883 | 0.873 | 1.906 | 328.6 |
| Attained height at 24 months (cm) | M3: + chart PAH (diagnostic) | 78 | 3.167 | 0.634 | [1.901, 4.433] | <0.001 | 0.884 | 0.873 | 1.907 | 329.6 |
| Change in height SDS, baseline to 24 months | M0: treatment only | 77 | 0.438 | 0.077 | [0.283, 0.592] | <0.001 | 0.299 | 0.289 | 0.340 | 54.2 |
| Change in height SDS, baseline to 24 months | M1: standard covariates | 77 | 0.534 | 0.100 | [0.334, 0.734] | <0.001 | 0.410 | 0.369 | 0.320 | 48.9 |
| Change in height SDS, baseline to 24 months | M2: + baseline height (primary) | 77 | 0.464 | 0.099 | [0.266, 0.663] | <0.001 | 0.461 | 0.415 | 0.308 | 43.9 |
| Change in height SDS, baseline to 24 months | M3: + chart PAH (diagnostic) | 77 | 0.459 | 0.102 | [0.255, 0.664] | <0.001 | 0.466 | 0.412 | 0.309 | 45.3 |

## S1c. Residual diagnostics and term-specific VIFs

| **Endpoint** | **Model** | **Breusch–Pagan p** | **Shapiro–Wilk p** | **Maximum Cook's D** | **4/n** | **Condition index** | **Maximum VIF** | **Term VIFs (first imputation)** |
| --- | --- | --- | --- | --- | --- | --- | --- | --- |
| Near-final adult height (cm) | M0 | 0.905 [0.905, 0.905] | 0.998 [0.998, 0.998] | 0.092 [0.092, 0.092] | 0.044 | 1.00 [1.00, 1.00] | 1.00 [1.00, 1.00] | treat=1.00 |
| Near-final adult height (cm) | M1 | 0.847 [0.510, 0.963] | 0.746 [0.495, 0.983] | 0.132 [0.096, 0.171] | 0.044 | 2.27 [1.97, 2.52] | 1.69 [1.49, 1.82] | treat=1.56; age=1.70; BMI=1.27; IGF-1=1.11; BA–CA=1.38 |
| Near-final adult height (cm) | M2 | 0.839 [0.531, 0.960] | 0.764 [0.436, 0.983] | 0.117 [0.088, 0.148] | 0.044 | 3.14 [2.87, 3.39] | 2.66 [2.28, 2.89] | treat=1.74; age=2.84; BMI=1.34; IGF-1=1.12; BA–CA=1.69; height=2.13 |
| Near-final adult height (cm) | M3 | 0.825 [0.476, 0.975] | 0.465 [0.171, 0.667] | 0.130 [0.102, 0.163] | 0.044 | 3.17 [2.93, 3.41] | 2.66 [2.28, 2.89] | treat=2.06; age=2.85; BMI=1.37; IGF-1=1.13; BA–CA=1.72; height=2.16; chart PAH=1.41 |
| Height gain, baseline to 24 months (cm) | M0 | 0.367 [0.367, 0.367] | 0.404 [0.404, 0.404] | 0.072 [0.072, 0.072] | 0.052 | 1.00 [1.00, 1.00] | 1.00 [1.00, 1.00] | treat=1.00 |
| Height gain, baseline to 24 months (cm) | M1 | 0.565 [0.337, 0.801] | 0.537 [0.287, 0.910] | 0.096 [0.086, 0.148] | 0.052 | 2.57 [2.38, 2.75] | 1.99 [1.87, 2.20] | treat=2.01; age=1.97; BMI=1.25; IGF-1=1.26; BA–CA=1.44 |
| Height gain, baseline to 24 months (cm) | M2 | 0.660 [0.385, 0.859] | 0.574 [0.280, 0.907] | 0.158 [0.118, 0.216] | 0.052 | 3.39 [3.24, 3.65] | 3.16 [2.93, 3.54] | treat=2.09; age=3.10; BMI=1.31; IGF-1=1.26; BA–CA=1.55; height=1.80 |
| Height gain, baseline to 24 months (cm) | M3 | 0.592 [0.321, 0.907] | 0.600 [0.289, 0.927] | 0.153 [0.116, 0.228] | 0.052 | 3.49 [3.26, 3.67] | 3.16 [2.98, 3.54] | treat=2.18; age=3.11; BMI=1.32; IGF-1=1.26; BA–CA=1.66; height=1.82; chart PAH=1.15 |
| Attained height at 24 months (cm) | M0 | 0.448 [0.448, 0.448] | 0.313 [0.313, 0.313] | 0.108 [0.108, 0.108] | 0.051 | 1.00 [1.00, 1.00] | 1.00 [1.00, 1.00] | treat=1.00 |
| Attained height at 24 months (cm) | M1 | 0.395 [0.153, 0.692] | 3.31e-04 [8.88e-05, 0.002] | 0.310 [0.266, 0.418] | 0.051 | 2.63 [2.47, 2.74] | 2.06 [1.92, 2.17] | treat=1.82; age=1.96; BMI=1.29; IGF-1=1.10; BA–CA=1.52 |
| Attained height at 24 months (cm) | M2 | 0.598 [0.291, 0.797] | 0.605 [0.222, 0.796] | 0.164 [0.085, 0.220] | 0.051 | 3.42 [3.24, 3.79] | 3.21 [2.90, 3.53] | treat=1.96; age=3.27; BMI=1.34; IGF-1=1.13; BA–CA=1.64; height=1.89 |
| Attained height at 24 months (cm) | M3 | 0.611 [0.350, 0.861] | 0.643 [0.179, 0.841] | 0.167 [0.130, 0.248] | 0.051 | 3.47 [3.24, 3.80] | 3.24 [2.90, 3.53] | treat=2.08; age=3.27; BMI=1.34; IGF-1=1.14; BA–CA=1.66; height=1.89; chart PAH=1.14 |
| Change in height SDS, baseline to 24 months | M0 | 0.439 [0.439, 0.439] | 0.141 [0.141, 0.141] | 0.072 [0.072, 0.072] | 0.052 | 1.00 [1.00, 1.00] | 1.00 [1.00, 1.00] | treat=1.00 |
| Change in height SDS, baseline to 24 months | M1 | 0.206 [0.063, 0.609] | 0.483 [0.213, 0.980] | 0.094 [0.055, 0.144] | 0.052 | 2.60 [2.39, 2.77] | 2.01 [1.89, 2.25] | treat=1.99; age=1.99; BMI=1.25; IGF-1=1.27; BA–CA=1.46 |
| Change in height SDS, baseline to 24 months | M2 | 0.154 [0.085, 0.537] | 0.808 [0.261, 0.971] | 0.139 [0.090, 0.254] | 0.052 | 3.39 [3.24, 3.64] | 3.15 [2.93, 3.54] | treat=2.08; age=3.10; BMI=1.31; IGF-1=1.27; BA–CA=1.54; height=1.78 |
| Change in height SDS, baseline to 24 months | M3 | 0.151 [0.028, 0.616] | 0.787 [0.253, 0.991] | 0.145 [0.085, 0.224] | 0.052 | 3.47 [3.25, 3.67] | 3.16 [2.97, 3.54] | treat=2.16; age=3.10; BMI=1.32; IGF-1=1.27; BA–CA=1.65; height=1.79; chart PAH=1.15 |

## S1d. Complete pooled regression coefficients

Each row is a pooled coefficient from the indicated model. Degrees of freedom use the Barnard–Rubin small-sample adjustment where between-imputation variance is present.

| **Endpoint** | **Model** | **Term** | **Coefficient** | **SE** | **df** | **95% CI** | **p** |
| --- | --- | --- | --- | --- | --- | --- | --- |
| Near-final adult height (cm) | M0 | Intercept | 160.1667 | 0.5412 | 89.0 | [159.091, 161.242] | <0.001 |
| Near-final adult height (cm) | M0 | Treatment (GnRHa + rhGH) | 1.7008 | 0.8163 | 89.0 | [0.079, 3.323] | 0.040 |
| Near-final adult height (cm) | M1 | Intercept | 161.9865 | 5.5454 | 77.2 | [150.945, 173.028] | <0.001 |
| Near-final adult height (cm) | M1 | Treatment (GnRHa + rhGH) | 2.2102 | 1.0130 | 79.2 | [0.194, 4.226] | 0.032 |
| Near-final adult height (cm) | M1 | Baseline age (years) | -0.3822 | 0.5869 | 75.4 | [-1.551, 0.787] | 0.517 |
| Near-final adult height (cm) | M1 | Baseline BMI (kg/m²) | 0.2593 | 0.2559 | 50.7 | [-0.255, 0.773] | 0.316 |
| Near-final adult height (cm) | M1 | Baseline IGF-1 (ng/mL) | -0.0073 | 0.0048 | 56.0 | [-0.017, 0.002] | 0.133 |
| Near-final adult height (cm) | M1 | Baseline BA–CA (years) | -0.4721 | 0.8023 | 58.1 | [-2.078, 1.134] | 0.559 |
| Near-final adult height (cm) | M2 | Intercept | 160.7096 | 10.3711 | 76.9 | [140.057, 181.362] | <0.001 |
| Near-final adult height (cm) | M2 | Treatment (GnRHa + rhGH) | 2.2592 | 1.0739 | 77.0 | [0.121, 4.398] | 0.039 |
| Near-final adult height (cm) | M2 | Baseline age (years) | -0.4450 | 0.7497 | 70.7 | [-1.940, 1.050] | 0.555 |
| Near-final adult height (cm) | M2 | Baseline BMI (kg/m²) | 0.2548 | 0.2598 | 51.4 | [-0.267, 0.776] | 0.331 |
| Near-final adult height (cm) | M2 | Baseline IGF-1 (ng/mL) | -0.0074 | 0.0048 | 55.1 | [-0.017, 0.002] | 0.132 |
| Near-final adult height (cm) | M2 | Baseline BA–CA (years) | -0.5205 | 0.9136 | 53.9 | [-2.352, 1.311] | 0.571 |
| Near-final adult height (cm) | M2 | Baseline height (cm) | 0.0147 | 0.1034 | 72.9 | [-0.191, 0.221] | 0.887 |
| Near-final adult height (cm) | M3 | Intercept | -4.9183 | 45.5487 | 77.2 | [-95.613, 85.777] | 0.914 |
| Near-final adult height (cm) | M3 | Treatment (GnRHa + rhGH) | 0.5788 | 1.0918 | 76.9 | [-1.595, 2.753] | 0.598 |
| Near-final adult height (cm) | M3 | Baseline age (years) | -0.4421 | 0.6891 | 72.3 | [-1.816, 0.931] | 0.523 |
| Near-final adult height (cm) | M3 | Baseline BMI (kg/m²) | 0.3002 | 0.2305 | 61.0 | [-0.161, 0.761] | 0.198 |
| Near-final adult height (cm) | M3 | Baseline IGF-1 (ng/mL) | -0.0063 | 0.0044 | 57.2 | [-0.015, 0.003] | 0.160 |
| Near-final adult height (cm) | M3 | Baseline BA–CA (years) | -0.9787 | 0.8279 | 61.6 | [-2.634, 0.676] | 0.242 |
| Near-final adult height (cm) | M3 | Baseline height (cm) | 0.0711 | 0.0963 | 74.2 | [-0.121, 0.263] | 0.463 |
| Near-final adult height (cm) | M3 | Chart-recorded baseline PAH (cm) | 0.9968 | 0.2674 | 77.6 | [0.464, 1.529] | <0.001 |
| Height gain, baseline to 24 months (cm) | M0 | Intercept | 10.9975 | 0.3304 | 75.0 | [10.339, 11.656] | <0.001 |
| Height gain, baseline to 24 months (cm) | M0 | Treatment (GnRHa + rhGH) | 2.2187 | 0.4766 | 75.0 | [1.269, 3.168] | <0.001 |
| Height gain, baseline to 24 months (cm) | M1 | Intercept | 21.8048 | 3.1515 | 61.2 | [15.503, 28.106] | <0.001 |
| Height gain, baseline to 24 months (cm) | M1 | Treatment (GnRHa + rhGH) | 3.3429 | 0.5908 | 66.7 | [2.164, 4.522] | <0.001 |
| Height gain, baseline to 24 months (cm) | M1 | Baseline age (years) | -1.1197 | 0.3053 | 64.2 | [-1.729, -0.510] | <0.001 |
| Height gain, baseline to 24 months (cm) | M1 | Baseline BMI (kg/m²) | 0.0276 | 0.1225 | 50.5 | [-0.218, 0.274] | 0.823 |
| Height gain, baseline to 24 months (cm) | M1 | Baseline IGF-1 (ng/mL) | -7.90e-04 | 0.0029 | 47.7 | [-0.007, 0.005] | 0.785 |
| Height gain, baseline to 24 months (cm) | M1 | Baseline BA–CA (years) | -0.9964 | 0.3681 | 54.0 | [-1.734, -0.258] | 0.009 |
| Height gain, baseline to 24 months (cm) | M2 | Intercept | 25.3896 | 5.9788 | 65.5 | [13.451, 37.329] | <0.001 |
| Height gain, baseline to 24 months (cm) | M2 | Treatment (GnRHa + rhGH) | 3.2270 | 0.6168 | 65.4 | [1.995, 4.459] | <0.001 |
| Height gain, baseline to 24 months (cm) | M2 | Baseline age (years) | -0.9597 | 0.3910 | 61.2 | [-1.741, -0.178] | 0.017 |
| Height gain, baseline to 24 months (cm) | M2 | Baseline BMI (kg/m²) | 0.0399 | 0.1245 | 49.7 | [-0.210, 0.290] | 0.750 |
| Height gain, baseline to 24 months (cm) | M2 | Baseline IGF-1 (ng/mL) | -5.64e-04 | 0.0029 | 46.4 | [-0.006, 0.005] | 0.848 |
| Height gain, baseline to 24 months (cm) | M2 | Baseline BA–CA (years) | -0.9369 | 0.3823 | 53.4 | [-1.704, -0.170] | 0.018 |
| Height gain, baseline to 24 months (cm) | M2 | Baseline height (cm) | -0.0395 | 0.0581 | 61.5 | [-0.156, 0.077] | 0.499 |
| Height gain, baseline to 24 months (cm) | M3 | Intercept | 18.0408 | 28.7445 | 42.8 | [-39.936, 76.018] | 0.534 |
| Height gain, baseline to 24 months (cm) | M3 | Treatment (GnRHa + rhGH) | 3.1994 | 0.6353 | 64.9 | [1.931, 4.468] | <0.001 |
| Height gain, baseline to 24 months (cm) | M3 | Baseline age (years) | -0.9680 | 0.3913 | 61.7 | [-1.750, -0.186] | 0.016 |
| Height gain, baseline to 24 months (cm) | M3 | Baseline BMI (kg/m²) | 0.0376 | 0.1269 | 47.9 | [-0.218, 0.293] | 0.769 |
| Height gain, baseline to 24 months (cm) | M3 | Baseline IGF-1 (ng/mL) | -4.89e-04 | 0.0030 | 45.5 | [-0.006, 0.006] | 0.870 |
| Height gain, baseline to 24 months (cm) | M3 | Baseline BA–CA (years) | -0.9531 | 0.3883 | 52.8 | [-1.732, -0.174] | 0.017 |
| Height gain, baseline to 24 months (cm) | M3 | Baseline height (cm) | -0.0384 | 0.0589 | 60.0 | [-0.156, 0.079] | 0.517 |
| Height gain, baseline to 24 months (cm) | M3 | Chart-recorded baseline PAH (cm) | 0.0461 | 0.1741 | 42.5 | [-0.305, 0.397] | 0.792 |
| Attained height at 24 months (cm) | M0 | Intercept | 144.6317 | 0.7655 | 76.0 | [143.107, 146.156] | <0.001 |
| Attained height at 24 months (cm) | M0 | Treatment (GnRHa + rhGH) | 4.3953 | 1.1114 | 76.0 | [2.182, 6.609] | <0.001 |
| Attained height at 24 months (cm) | M1 | Intercept | 112.4469 | 7.0112 | 66.2 | [98.449, 126.445] | <0.001 |
| Attained height at 24 months (cm) | M1 | Treatment (GnRHa + rhGH) | 0.4834 | 1.3390 | 66.9 | [-2.189, 3.156] | 0.719 |
| Attained height at 24 months (cm) | M1 | Baseline age (years) | 2.9771 | 0.7036 | 64.5 | [1.572, 4.383] | <0.001 |
| Attained height at 24 months (cm) | M1 | Baseline BMI (kg/m²) | 0.3354 | 0.2866 | 43.8 | [-0.242, 0.913] | 0.248 |
| Attained height at 24 months (cm) | M1 | Baseline IGF-1 (ng/mL) | 0.0041 | 0.0065 | 44.6 | [-0.009, 0.017] | 0.529 |
| Attained height at 24 months (cm) | M1 | Baseline BA–CA (years) | 0.5788 | 0.8592 | 48.2 | [-1.148, 2.306] | 0.504 |
| Attained height at 24 months (cm) | M2 | Intercept | 25.7990 | 5.9142 | 67.0 | [13.994, 37.604] | <0.001 |
| Attained height at 24 months (cm) | M2 | Treatment (GnRHa + rhGH) | 3.2439 | 0.6103 | 66.2 | [2.025, 4.462] | <0.001 |
| Attained height at 24 months (cm) | M2 | Baseline age (years) | -0.9383 | 0.3975 | 59.2 | [-1.734, -0.143] | 0.022 |
| Attained height at 24 months (cm) | M2 | Baseline BMI (kg/m²) | 0.0571 | 0.1272 | 43.8 | [-0.199, 0.314] | 0.656 |
| Attained height at 24 months (cm) | M2 | Baseline IGF-1 (ng/mL) | -0.0010 | 0.0030 | 38.2 | [-0.007, 0.005] | 0.734 |
| Attained height at 24 months (cm) | M2 | Baseline BA–CA (years) | -0.9106 | 0.3854 | 50.1 | [-1.685, -0.137] | 0.022 |
| Attained height at 24 months (cm) | M2 | Baseline height (cm) | 0.9546 | 0.0583 | 59.1 | [0.838, 1.071] | <0.001 |
| Attained height at 24 months (cm) | M3 | Intercept | 10.5430 | 26.8878 | 53.2 | [-43.383, 64.469] | 0.697 |
| Attained height at 24 months (cm) | M3 | Treatment (GnRHa + rhGH) | 3.1670 | 0.6337 | 63.8 | [1.901, 4.433] | <0.001 |
| Attained height at 24 months (cm) | M3 | Baseline age (years) | -0.9488 | 0.3996 | 58.4 | [-1.748, -0.149] | 0.021 |
| Attained height at 24 months (cm) | M3 | Baseline BMI (kg/m²) | 0.0546 | 0.1295 | 41.7 | [-0.207, 0.316] | 0.676 |
| Attained height at 24 months (cm) | M3 | Baseline IGF-1 (ng/mL) | -9.17e-04 | 0.0030 | 37.4 | [-0.007, 0.005] | 0.764 |
| Attained height at 24 months (cm) | M3 | Baseline BA–CA (years) | -0.9298 | 0.3924 | 48.5 | [-1.719, -0.141] | 0.022 |
| Attained height at 24 months (cm) | M3 | Baseline height (cm) | 0.9578 | 0.0590 | 58.1 | [0.840, 1.076] | <0.001 |
| Attained height at 24 months (cm) | M3 | Chart-recorded baseline PAH (cm) | 0.0943 | 0.1620 | 53.4 | [-0.231, 0.419] | 0.563 |
| Change in height SDS, baseline to 24 months | M0 | Intercept | -0.2416 | 0.0537 | 75.0 | [-0.349, -0.135] | <0.001 |
| Change in height SDS, baseline to 24 months | M0 | Treatment (GnRHa + rhGH) | 0.4379 | 0.0775 | 75.0 | [0.283, 0.592] | <0.001 |
| Change in height SDS, baseline to 24 months | M1 | Intercept | 0.9609 | 0.5388 | 59.1 | [-0.117, 2.039] | 0.080 |
| Change in height SDS, baseline to 24 months | M1 | Treatment (GnRHa + rhGH) | 0.5345 | 0.1001 | 65.5 | [0.334, 0.734] | <0.001 |
| Change in height SDS, baseline to 24 months | M1 | Baseline age (years) | -0.1062 | 0.0522 | 62.7 | [-0.211, -0.002] | 0.046 |
| Change in height SDS, baseline to 24 months | M1 | Baseline BMI (kg/m²) | 0.0063 | 0.0204 | 52.7 | [-0.035, 0.047] | 0.760 |
| Change in height SDS, baseline to 24 months | M1 | Baseline IGF-1 (ng/mL) | -4.11e-04 | 5.02e-04 | 42.4 | [-0.001, 6.01e-04] | 0.417 |
| Change in height SDS, baseline to 24 months | M1 | Baseline BA–CA (years) | -0.1715 | 0.0634 | 52.6 | [-0.299, -0.044] | 0.009 |
| Change in height SDS, baseline to 24 months | M2 | Intercept | 3.1100 | 0.9598 | 65.8 | [1.194, 5.026] | 0.002 |
| Change in height SDS, baseline to 24 months | M2 | Treatment (GnRHa + rhGH) | 0.4645 | 0.0994 | 65.2 | [0.266, 0.663] | <0.001 |
| Change in height SDS, baseline to 24 months | M2 | Baseline age (years) | -0.0103 | 0.0631 | 60.9 | [-0.136, 0.116] | 0.871 |
| Change in height SDS, baseline to 24 months | M2 | Baseline BMI (kg/m²) | 0.0140 | 0.0198 | 52.4 | [-0.026, 0.054] | 0.484 |
| Change in height SDS, baseline to 24 months | M2 | Baseline IGF-1 (ng/mL) | -2.84e-04 | 4.77e-04 | 44.8 | [-0.001, 6.77e-04] | 0.555 |
| Change in height SDS, baseline to 24 months | M2 | Baseline BA–CA (years) | -0.1374 | 0.0631 | 50.7 | [-0.264, -0.011] | 0.034 |
| Change in height SDS, baseline to 24 months | M2 | Baseline height (cm) | -0.0237 | 0.0092 | 63.5 | [-0.042, -0.005] | 0.012 |
| Change in height SDS, baseline to 24 months | M3 | Intercept | 1.7408 | 4.7562 | 38.7 | [-7.882, 11.364] | 0.716 |
| Change in height SDS, baseline to 24 months | M3 | Treatment (GnRHa + rhGH) | 0.4591 | 0.1024 | 64.4 | [0.255, 0.664] | <0.001 |
| Change in height SDS, baseline to 24 months | M3 | Baseline age (years) | -0.0118 | 0.0631 | 61.5 | [-0.138, 0.114] | 0.852 |
| Change in height SDS, baseline to 24 months | M3 | Baseline BMI (kg/m²) | 0.0137 | 0.0202 | 49.9 | [-0.027, 0.054] | 0.500 |
| Change in height SDS, baseline to 24 months | M3 | Baseline IGF-1 (ng/mL) | -2.70e-04 | 4.85e-04 | 43.6 | [-0.001, 7.08e-04] | 0.580 |
| Change in height SDS, baseline to 24 months | M3 | Baseline BA–CA (years) | -0.1405 | 0.0641 | 50.0 | [-0.269, -0.012] | 0.033 |
| Change in height SDS, baseline to 24 months | M3 | Baseline height (cm) | -0.0234 | 0.0093 | 61.7 | [-0.042, -0.005] | 0.014 |
| Change in height SDS, baseline to 24 months | M3 | Chart-recorded baseline PAH (cm) | 0.0086 | 0.0288 | 38.5 | [-0.050, 0.067] | 0.768 |

# Table S2. Stepwise coefficient changes and sensitivity analyses

## S2a. Sequential covariate entry within a fixed endpoint sample

| **Endpoint** | **Step** | **n** | **Treatment estimate** | **SE** | **95% CI** | **p** | **Change from previous step** |
| --- | --- | --- | --- | --- | --- | --- | --- |
| Near-final adult height (cm) | S0 treatment only | 91 | 1.701 | 0.816 | [0.079, 3.323] | 0.040 | — |
| Near-final adult height (cm) | S1 + baseline age | 91 | 2.020 | 0.950 | [0.131, 3.908] | 0.036 | 0.319 |
| Near-final adult height (cm) | S2 + BMI | 91 | 2.170 | 0.993 | [0.195, 4.145] | 0.032 | 0.150 |
| Near-final adult height (cm) | S3 + IGF-1 | 91 | 2.089 | 0.987 | [0.126, 4.052] | 0.037 | -0.081 |
| Near-final adult height (cm) | S4 + BA-CA (= M1) | 91 | 2.210 | 1.013 | [0.194, 4.226] | 0.032 | 0.121 |
| Near-final adult height (cm) | S5 + baseline height (= M2) | 91 | 2.259 | 1.074 | [0.121, 4.398] | 0.039 | 0.049 |
| Near-final adult height (cm) | S6 + chart-recorded PAH (= M3) | 91 | 0.579 | 1.092 | [-1.595, 2.753] | 0.598 | -1.680 |
| Height gain, baseline to 24 months (cm) | S0 treatment only | 77 | 2.219 | 0.477 | [1.269, 3.168] | <0.001 | — |
| Height gain, baseline to 24 months (cm) | S1 + baseline age | 77 | 3.309 | 0.597 | [2.119, 4.498] | <0.001 | 1.090 |
| Height gain, baseline to 24 months (cm) | S2 + BMI | 77 | 3.307 | 0.598 | [2.114, 4.500] | <0.001 | -0.002 |
| Height gain, baseline to 24 months (cm) | S3 + IGF-1 | 77 | 3.337 | 0.614 | [2.113, 4.561] | <0.001 | 0.030 |
| Height gain, baseline to 24 months (cm) | S4 + BA-CA (= M1) | 77 | 3.343 | 0.591 | [2.164, 4.522] | <0.001 | 0.006 |
| Height gain, baseline to 24 months (cm) | S5 + baseline height (= M2) | 77 | 3.227 | 0.617 | [1.995, 4.459] | <0.001 | -0.116 |
| Height gain, baseline to 24 months (cm) | S6 + chart-recorded PAH (= M3) | 77 | 3.199 | 0.635 | [1.931, 4.468] | <0.001 | -0.028 |
| Attained height at 24 months (cm) | S0 treatment only | 78 | 4.395 | 1.111 | [2.182, 6.609] | <0.001 | — |
| Attained height at 24 months (cm) | S1 + baseline age | 78 | 0.653 | 1.310 | [-1.958, 3.263] | 0.620 | -3.743 |
| Attained height at 24 months (cm) | S2 + BMI | 78 | 0.650 | 1.297 | [-1.935, 3.236] | 0.618 | -0.003 |
| Attained height at 24 months (cm) | S3 + IGF-1 | 78 | 0.493 | 1.329 | [-2.160, 3.145] | 0.712 | -0.157 |
| Attained height at 24 months (cm) | S4 + BA-CA (= M1) | 78 | 0.483 | 1.339 | [-2.189, 3.156] | 0.719 | -0.009 |
| Attained height at 24 months (cm) | S5 + baseline height (= M2) | 78 | 3.244 | 0.610 | [2.025, 4.462] | <0.001 | 2.760 |
| Attained height at 24 months (cm) | S6 + chart-recorded PAH (= M3) | 78 | 3.167 | 0.634 | [1.901, 4.433] | <0.001 | -0.077 |
| Change in height SDS, baseline to 24 months | S0 treatment only | 77 | 0.438 | 0.077 | [0.283, 0.592] | <0.001 | — |
| Change in height SDS, baseline to 24 months | S1 + baseline age | 77 | 0.512 | 0.101 | [0.310, 0.714] | <0.001 | 0.074 |
| Change in height SDS, baseline to 24 months | S2 + BMI | 77 | 0.512 | 0.101 | [0.309, 0.714] | <0.001 | -9.54e-05 |
| Change in height SDS, baseline to 24 months | S3 + IGF-1 | 77 | 0.527 | 0.103 | [0.321, 0.733] | <0.001 | 0.015 |
| Change in height SDS, baseline to 24 months | S4 + BA-CA (= M1) | 77 | 0.534 | 0.100 | [0.334, 0.734] | <0.001 | 0.008 |
| Change in height SDS, baseline to 24 months | S5 + baseline height (= M2) | 77 | 0.464 | 0.099 | [0.266, 0.663] | <0.001 | -0.070 |
| Change in height SDS, baseline to 24 months | S6 + chart-recorded PAH (= M3) | 77 | 0.459 | 0.102 | [0.255, 0.664] | <0.001 | -0.005 |

The attained-height estimate changes most when baseline height enters at S5: +0.48 cm at S4 (M1) to +3.24 cm at S5 (M2). Height gain and change in height SDS do not show this reversal. Because the endpoint sample remains fixed within each block, these changes reflect covariate entry rather than changes in analyzed patients.

## S2b. Complete-case and NFAH-criterion sensitivity analyses

| **Endpoint** | **Analysis** | **n** | **Treatment estimate** | **SE** | **95% CI** | **p** |
| --- | --- | --- | --- | --- | --- | --- |
| NFAH | 20-imputation M2 (primary) | 91 | 2.259 | 1.074 | [0.121, 4.398] | 0.039 |
| NFAH | Complete-case M2 with IGF-1 | 45 | 3.695 | 1.578 | [0.500, 6.891] | 0.025 |
| NFAH | Complete-case M2 without IGF-1 | 60 | 4.344 | 1.267 | [1.804, 6.884] | 0.001 |
| NFAH | M2 excluding criterion E | 89 | 2.105 | 1.065 | [-0.018, 4.227] | 0.052 |
| NFAH | M2 restricted to criteria A–C | 83 | 1.726 | 1.110 | [-0.487, 3.940] | 0.124 |
| 24-month height gain | 20-imputation M2 (primary) | 77 | 3.227 | 0.617 | [1.995, 4.459] | <0.001 |
| 24-month height gain | Complete-case M2 with IGF-1 | 36 | 2.443 | 0.856 | [0.694, 4.193] | 0.008 |
| 24-month height gain | Complete-case M2 without IGF-1 | 53 | 3.433 | 0.675 | [2.075, 4.791] | <0.001 |
